# Tolman's Sunburst Maze 80 Years on: A Meta‐Analysis Reveals Poor Replicability and Little Evidence for Shortcutting

**DOI:** 10.1111/ejn.70365

**Published:** 2026-01-05

**Authors:** Éléonore Duvelle, Roddy M. Grieves

**Affiliations:** ^1^ University of Glasgow School of Psychology and Neuroscience Glasgow UK

**Keywords:** cognition, navigation, shortcut, spatial, Tolman

## Abstract

The Sunburst maze, first described 80 years ago by Tolman, Ritchie and Kalish (1946) and popularized by Tolman (1948), is widely regarded as a classic demonstration of cognitive map use in rats. In this task, animals trained on a circuitous path to a reward were presented with new paths, including a shortcut, after the original route was blocked. A substantial proportion of rats selected the shortcut, which Tolman et al. (1946; 1948) interpreted as evidence that animals have an internal spatial representation, or ‘cognitive map’. Despite the influence of this study, attempts to replicate it have been largely unsuccessful. This review critically examines a dozen replications involving rats, squirrel monkeys and humans, highlighting a range of alternative strategies, with only a fraction of experiments demonstrating shortcutting (17%). Instead, most studies found that animals either favoured paths adjacent to the original training route (32%), did not have a preference (26%), chose unremarkable paths (13%) or selected options consistent with previously rewarded responses (6%), suggesting a reliance on procedural or associative learning rather than demonstrating flexible spatial inference. Although the original experiment has been widely criticized for including a visual cue above the reward location, subsequent studies rarely found that this feature guided path choices (6%). Neurophysiological data from hippocampal lesion and head‐direction cell studies further undermine the claim that shortcutting in the Sunburst maze depends on cognitive maps. We argue that this study, though historically significant, is a poor standalone demonstration of map‐based navigation.

## Graphical abstract

In 1946, Tolman et al. reported that rats could take a novel shortcut to a goal after training on an indirect route, supporting the Cognitive Map theory. However, a review of subsequent Sunburst maze studies shows this is an outlier: shortcutting has been observed in only a relatively small fraction of experiments. Instead, animals most often pick adjacent or unremarkable paths, pointing to response‐based strategies or novelty‐related confusion rather than map‐based spatial reasoning.

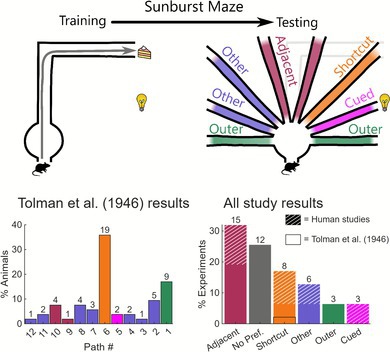

**Abbreviations**
A: ambient light
L‐BM: light underwent big move before testing
L‐L: stable light
L‐SML: light underwent small move right or left before testing
L‐SMR: light underwent small move right before testing

## 1 Introduction

**FIGURE 1:**
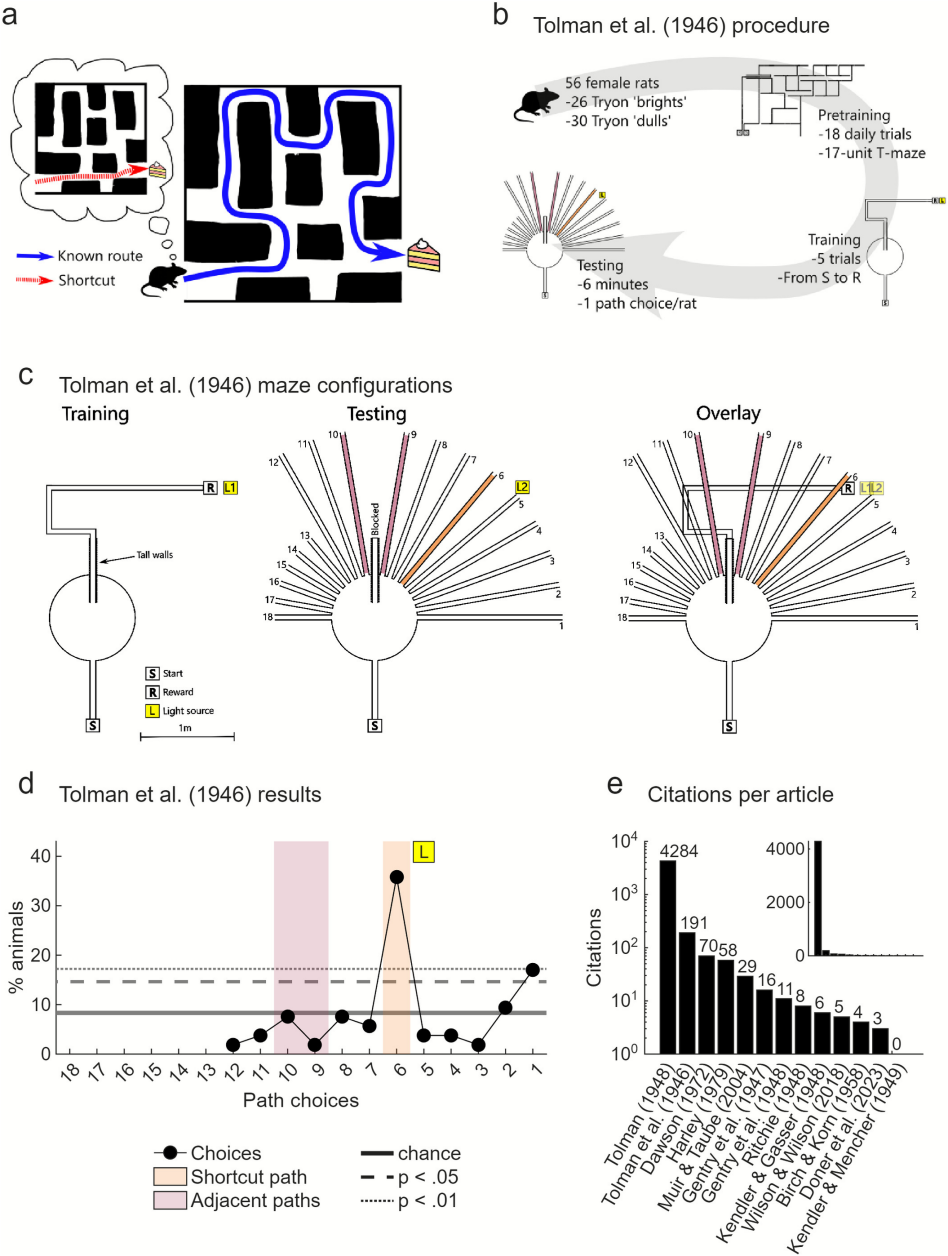
Shortcutting in the Sunburst maze. (a) Schematic representation of a shortcut. (b) Tolman et al. (1946) experiment procedure (for details of ‘Tryon’ rats or pretraining in the Tryon maze, see Sections 3.2, 3.3 and Figure S1). (c) Tolman et al. (1946) maze configurations. Based on data/description from Tolman et al. (1946). See their Figure 1 and Figure 2 (p. 16–17). Note that while the light source is described in‐text as being in the same position between training and testing, in their figures it changes position slightly relative to the maze; for consistency, we have reproduced these positions here (L1 for training and L2 for testing). (d) First path choices made during testing as a percentage of total. Values taken from Tolman et al.'s (1946) Figure 3 (p. 19). Note that Paths 13–18 were excluded from analyses, are only shown for consistency and are not used when computing chance level (see Methods: *Random path choice reallocation*). (e) The number of citations of Tolman et al.'s (1946) original report, Tolman's (1948) review article that popularized the experiment, and the subsequent replication attempts, log scale. Inset shows the same but with a linear scale. Citation metrics retrieved from Scopus.com as of May 2025. More recent experiments are expected to have accumulated fewer citations, but studies published contemporary to the original are also under cited (Gentry et al. 1947; Ritchie 1948).

Navigation is fundamental to survival—animals, including humans, must be able to locate food, shelter and mates across environments that are often unknown or changeable. In everyday life, much of this navigation relies on familiar, well‐learned routes. Yet, a central premise in spatial cognition is that animals can also navigate *flexibly*; they can reach a goal even when it is not directly visible, when the usual route is blocked, and when starting from an unfamiliar location. This form of flexible behaviour is widely thought to depend on a ‘cognitive map’: an internal representation of places, and of the relationships between them, that is independent of the animal's current position (O'Keefe and Nadel 1978; Poucet 1993; Arleo and Rondi‐Reig 2007). One of the most compelling tests of such a map is an animal's ability to infer novel shortcuts between known locations (O'Keefe and Nadel 1978; Bennett 1996; Healy et al. 2003; Menzel 2018) (Figure 1a). Yet, while the idea of cognitive mapping is foundational, empirical evidence for de novo shortcutting remains mixed (Bennett 1996; Grieves and Dudchenko 2013). Much of this debate can be traced back to Tolman et al.'s (1946) seminal ‘Sunburst’ maze experiment. In this study, rats were first trained to follow an indirect route to food. After training, they were presented with a ‘Sunburst’ array of novel paths, and the training route was blocked. Rats preferentially chose the path leading directly towards the previously rewarded location, seemingly inferring an optimal shortcut. Tolman interpreted this as evidence that rats had learned a ‘wider comprehensive map’ of space (Tolman 1948, 204), arguing that rats must have acquired latent map‐like spatial knowledge during training and, thus, that they do not navigate by simple stimulus–response associations.

Despite significant contemporary (Wilcoxon and Waters 1948; Kendler 1952; Estes et al. 1954, 179; Hilgard and Marquis 1961, 224) and later criticism (Woodworth 1971, 632; O'Keefe and Nadel 1978, 71; Olton 1979, 590; Thinus‐Blanc 1996, 10; Jensen 2006; Delmore 2024, 455), Tolman et al.'s (1946) experiment holds a privileged place in the history of psychology. Widely cited as foundational evidence for cognitive mapping, its findings have been integrated into textbooks, lectures and theoretical frameworks for decades (Haggbloom et al. 2002; Horner et al. 2025). The study's long‐standing prominence in the literature reflects its important contribution to the field. Yet, researchers have struggled to consistently replicate its most celebrated finding: shortcutting. The history of science reminds us that even the most influential findings can benefit from periodic re‐evaluation and reinterpretation. This review does not seek to dismiss Tolman's contribution but to contextualize it—exploring why these results have been so enduring, but difficult to replicate. By examining the full body of evidence, we can appreciate the Sunburst study's role in shaping the field while acknowledging that its legacy, like all scientific knowledge, remains open to refinement.

In this review, we revisit Tolman et al.'s (1946) experiment, examine the methodological issues that shaped its interpretation and compare its results to subsequent replication studies. To our knowledge, this is the first comprehensive synthesis of all Sunburst maze replications, providing a clearer understanding of what this classic task reveals about spatial navigation.

## 2 The Sunburst Maze

In the 1940s, a series of experiments by Edward Chase Tolman challenged the prevailing behaviourist view that animal learning consisted solely of stimulus–response associations. Through a series of studies entitled ‘Studies in spatial learning’, Tolman argued that rats form ‘expectancies’ that allow them to navigate flexibly rather than simply repeat reinforced behaviours (Tolman 1951). This theory laid the foundation for what he would later call a ‘cognitive map’ (Tolman 1948; for a review see Dudchenko 2010). The Sunburst maze was conceived as one of the critical tests of this idea: If rats learn a map of their environment, they should be able to infer a novel shortcut to a goal when their familiar path is blocked, whereas a purely response‐based account would predict failure in this situation.

Tolman et al.'s (1946) Sunburst experiment came in two parts: training followed by testing (Figure 1b,c). Fifty‐six female rats were first trained to traverse a central platform, then a long, elevated runway, comprising right‐angled turns forming an ‘S’ bend to reach a reward location. A desk lamp was placed behind the reward and pointed towards the last segment of the training path. This was the only source of illumination in the room and the only explicit visual cue, beyond the maze itself. The rats were trained incrementally and by the test day, all rats had completed five trials from the start point to the reward. For the shortcut test, the reward box was removed and the training path was blocked a short distance from the central platform. A new radial, ‘Sunburst’ arrangement of 18 equally spaced paths was available from this choice point (Figure 1c) (Tolman 1948). Crucially, one of these paths was angled so that it led very closely to the location of reward during training (Path #6). Each rat was given one test in the new maze configuration, and if a rat ran to the end of a pathway within 6 min, this was recorded as the rat's choice of path. The six leftmost paths were excluded, and shorter excursions of up to 60 cm were not recorded; however, we do know that rats explored ‘practically all the radiating paths’ (Tolman 1948, 204), ‘*all* of the rats’ explored the blocked training path first (Ritchie 1948, 661, emphasis his; see also Tolman et al. 1946, 18) and often returned to the start point (Tolman et al. 1946, 18).

The results of the Sunburst test were remarkable: 36% (19/53) of the rats exhibited a final choice preference for Path #6, the shortcut route (Figure 1d). The next most frequently chosen path was #1, the most eastward facing one, with 17% (9/53) of the visits. The other paths were chosen at or very close to chance level. From this, Tolman et al. (1946) concluded that the animals choosing the shortcut path had learned the location of the reward, acquired an expectation of reward at that location, and in the test trial inferred that Path #6 was the shortest route to this location. This line of reasoning would form a large foundation of support for Tolman's theory of expectancy moving forward (Tolman 1948, 1949, 1951, 1955; Tolman and Postman 1954; Amundson 1986). In anticipation of later criticism, Tolman et al. (1946) argued that the rats that did not choose the shortcut likely would have, if given more training and opportunity to learn the location of the reward. They also argued (without explicitly testing it, see Section 4.2) that the single light source used was an important cue of the reward location which was essential for the rats to make their spatial inference but did not act as a stimulus that the rats simply approached during testing.

**FIGURE 2:**
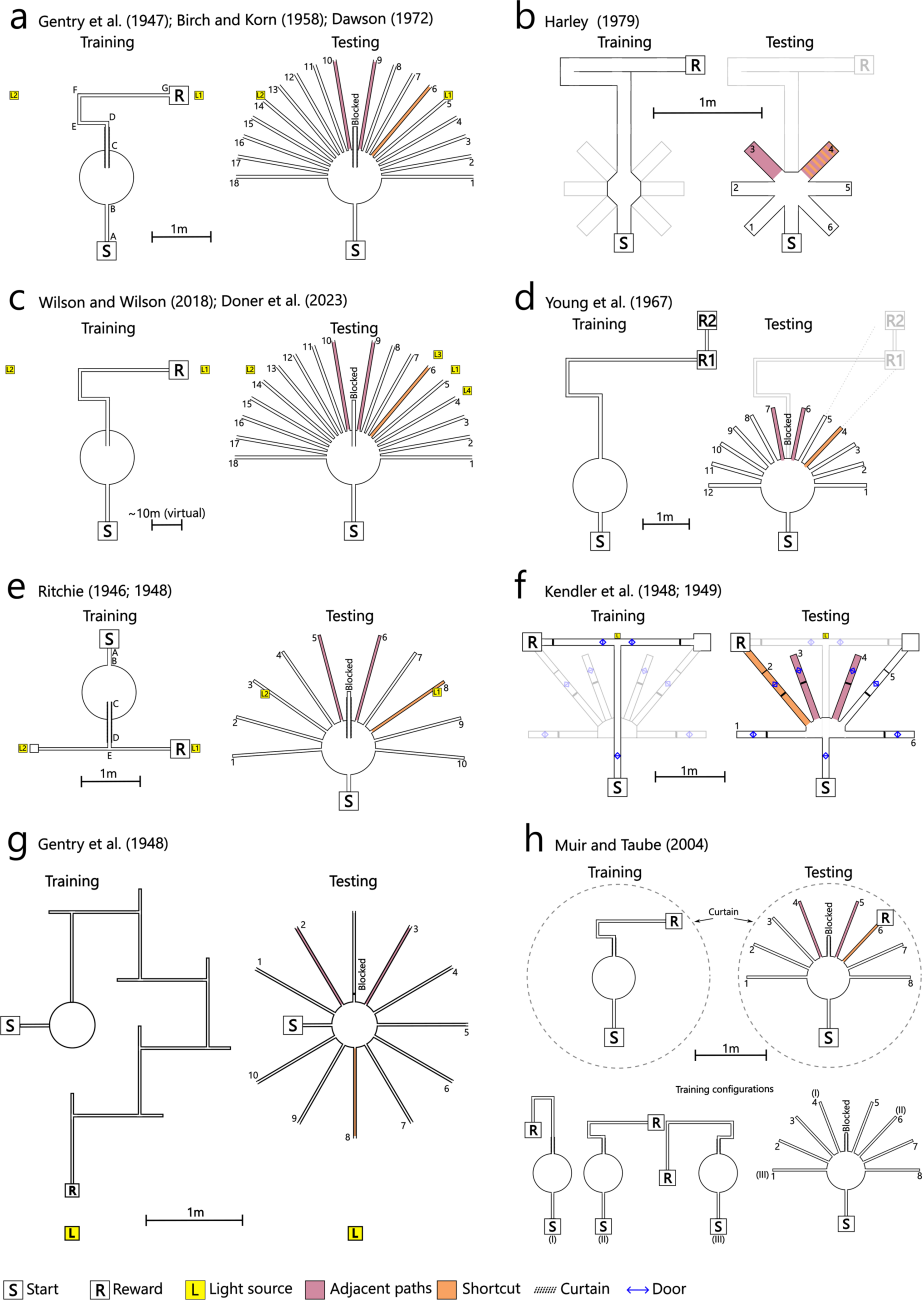
Variations of the Sunburst maze used in subsequent studies. For a detailed explanation of each protocol and results, see Figure S2–Figure S9. Based on data/description in the original texts. (a) Gentry et al. (1947), see also their Figure 2. Birch and Korn (1958), Dawson (1972) and Michik (1973) used a similar design, with only the former using shortened left arms as in Tolman et al. (1946). See also Figure S2 and Figure S10. (b) Harley (1979); see also her Figure 1. See also Figure S3. (c) Wilson and Wilson (2018); see also their Figure 1; Doner et al. (2023); see their Figure 1 and text. See also Figure S4. (d) Young et al. (1967), see also their Figure 1. See also Figure S5. (e) Ritchie (1948), and see also his Figures 3 and 4. Variations also used by Ritchie (1946 unpublished thesis). See also Figure S6. (f) Kendler and Gasser (1948), and see also their Figure 1. Also used by Kendler and Mencher (1949) and Chamberlain (1947 unpublished thesis). See also Figure S7. (g) Gentry et al. (1948), and see also their Figures 1 and 2. See also Figure S8. (h) Muir and Taube (2004), and see also their Figure 1. See also Figure S9.

Tolman et al.'s (1946) groundbreaking findings, and their interpretation, sparked widespread interest and prompted numerous research groups to explore the phenomenon further through various replication attempts (Figure 2a–h, see Figure S2–Figure S9 for detailed schematics and results). Surprisingly, across the 13 published studies (including the original) describing 47 experiments, shortcutting behaviour was above chance only in 17% of experiments (Tolman et al. 1946; Gentry et al. 1947, 1948; Kendler and Gasser 1948; Ritchie 1948; Kendler and Mencher 1949; Birch and Korn 1958; Young et al. 1967; Dawson 1972; Harley 1979; Muir and Taube 2004; Wilson and Wilson 2018; Doner et al. 2023). Additionally, replication attempts were reported in three graduate theses (Ritchie 1946; Chamberlain 1947; Michik 1973); in some cases, these overlap with published reports, but where they provide unique insights we discuss them here. Gaulin and FitzGerald (1986) used the same maze, but because path choices were not reported, they are not included in the present review work. Out of the experiments that positively replicated the shortcutting effect, one was based on a small group of four blind rats (Young et al. 1967), another was not able to replicate the effect later in the same study (Harley 1979) (discussed more in Section 5.2) and two further studies did not provide choice frequencies, making interpretation difficult (Dawson 1972; Michik 1973). The remaining positive replications were conducted using humans and after detailed testing ultimately concluded that the subjects strongly based their behaviour on the position of a visual cue, rather than inferring a shortcut (Wilson and Wilson 2018; Doner et al. 2023) (discussed more in Sections 4.3 and 4.4).

These studies are vastly under cited and overlooked when compared to the original study (Figure 1e): the original Sunburst study (Tolman et al. 1946, 191 citations) is cited almost three times more than the most cited replication attempt (Dawson 1972, 70 citations), with six of the 12 published replication attempts cited less than 10 times (Figure 1e). By comparison, the most impactful work of Tolman, *Cognitive Maps in Rats and Men* (Tolman 1948), which builds on the Sunburst results as one of the arguments supporting the cognitive map theory, has been cited 4284 times to date. We believe a better knowledge of these overlooked replication studies will lead to a more appropriately nuanced interpretation of Tolman's theories that rely on spatial inference demonstrations.

## 3 Methodological Limitations of the Original Study

Why did subsequent replications struggle to find a shortcutting effect when Tolman et al. (1946) reported such a high rate of shortcut use? To understand this discrepancy, it is important to first consider the methodological quirks and potential confounds of the original study. Some of these factors directly weaken the strength of their interpretation, while others have simply made close replication more difficult (see Table S1 for a complete summary). However, to date, neither individual methodological factors nor their combination satisfactorily explain why Tolman et al.'s (1946) findings differ from those of later studies, including those that closely reproduced key elements of the original protocol.

### 3.1 Scoring Committed Choices

In the original study, a choice was scored only when a rat ran all the way to the end of a path (100% of the 1.83 m long paths: Tolman et al. 1946). This lenient criterion allowed a lot of deliberative and exploratory behaviour prior to making a final path selection; indeed, the mean choice time was 3.5 min and ‘No rat made any choice without having first gone around the edge of the table‐top at least once, and without having tentatively explored more than one other path’ (p. 18). While several subsequent studies adopted stricter criteria for a rat's ‘final choice’ that would permit only shorter explorations (80% of 1.5 m: Ritchie 1946, 1948; 50% of 1.5 m: Kendler and Gasser 1948; Kendler and Mencher 1949), others used similar criteria (100% of 1.83 m: Gentry et al. 1947, 100% of 0.92 m: Gentry et al. 1948; 100% of 1.83 m: Birch and Korn 1958; 100% of 0.92 m: Muir and Taube 2004; 100% of 64 m virtual paths: Doner et al. 2023), or less clearly defined measures (undefined: Young et al. 1967; 30% of unknown length paths: Wilson and Wilson 2018). Harley's (1979) maze was much smaller in scale than these other studies; rats in this case had to run to the end of 0.4 m long paths, but they had to fully pass through a curtain placed at the end of the arm for their choice to be scored. Interestingly, Dawson (1972), replicating Tolman et al.'s (1946) maze design, defined a choice as a rat entering 0.3 m along the 1.83 m paths because ‘the criterion Tolman used […] was considered to be too difficult’ (p. 32) (see also Michik 1973). These definitional inconsistencies could significantly impact how behaviour is interpreted, although the studies replicating Tolman et al.'s (1946) criteria still did not observe shortcutting behaviour.

Tolman et al.'s (1946) scoring also excluded responses to the leftmost arms of the maze, apparently post hoc, on the basis that those arms were shorter than the others (the maze could not fit within the experiment room with all of the arms full‐length). These arms headed away from the reward location, biasing the remaining choices towards the reward location. The rats that chose one of these shorter arms were not excluded from the study; instead their second choice was included in the final results, mixing first‐ and second‐order choices. No subsequent replication adopted this asymmetric design except for Birch and Korn (1958), and it remains unclear if the peculiarities of Tolman et al.'s (1946) apparatus and scoring conventions inflated the apparent preference for the shortcut.

### 3.2 Rat Strain, Age and Sex

Another critical difference between Tolman et al. (1946) and subsequent studies lies in the subjects used, which were ‘Tryon’ rats (S_1_ and S_3_ strains), selectively bred based on their maze‐solving performance (Tryon 1929, see Tryon 1930, 161 for strain details, Tryon 1931). The two strains were divided according to their low or high error rates (for ‘brights’ or ‘dull’ rats, respectively) in a 17‐unit multiple T‐maze (reviewed by Innis 1992) (Figure 1b and Figure S1). In modern classification, this task would probably be considered a sequential egocentric task that rats can solve by learning a succession of left or right turns (Arleo and Rondi‐Reig 2007), or perhaps an odour‐based task, as rats could follow the odour trails left by previous rats to reach the exit (see also Rosenberg et al. 2021). Neither of these strategies are map‐based, meaning that they do not rely on a cognitive map. Regardless of whether ‘maze‐solving’ ability can be genetically transmitted, it is thus unclear whether ‘bright’ rats should have been better at spatial inference than ‘dull’ rats, and in fact, Tolman et al. (1946) found that a roughly equal number of ‘dull’ and ‘bright’ rats chose the shortcut path (discussed more in Section 5). Krechevsky (1933) and Searle (1949) reported that Tryon's rats expressed significantly different behaviour to contemporary stock animals in terms of visual discrimination, rearing behaviour, navigation strategies and motivation. No subsequent replication study procured these rat strains, leaving open the possibility that the Tryon rats demonstrated unusual responses in the Sunburst task. In his later Sunburst maze experiments, Ritchie (1946, 1948) did use ‘MxM’ rats: A median strain bred from Tryon's ‘bright’ and ‘dull’ strains. This was the closest replication of Tolman et al.'s (1946) study in terms of the animals used, but it did not report significant shortcutting behaviour.

Although Tolman et al. (1946) did not specify the pigmentation of their rats, based on contemporary studies, it is likely that most, if not all, of the rats in Tolman's experiment were pigmented (Krechevsky 1933; Ritchie 1948; Searle 1949). In contrast, the majority of replication studies in rats have used albino strains (Chamberlain 1947; Gentry et al. 1947, 1948; Kendler and Gasser 1948; Kendler and Mencher 1949; Birch and Korn 1958; Young et al. 1967; Harley 1979) (Table S1). This is noteworthy because albino rat strains generally show reduced visual acuity and altered light sensitivity compared with pigmented strains (Lashley 1930; Restle 1957; Prusky et al. 2002), which could reduce their effective use of distal visual landmarks in navigation tasks (but see Harker and Whishaw 2002; Gökçek‐Saraç et al. 2015). However, studies that did use pigmented strains (Ritchie 1946, 1948; Gentry et al. 1948; Muir and Taube 2004), human participants (Wilson and Wilson 2018; Doner et al. 2023) or non‐human primates (Young et al. 1967) largely did not observe shortcutting, or found that when it occurred, it was due to beaconing to the light instead of choosing the shortcut. Thus, while rat strain and pigmentation may partially account for the variability in results, neither are likely to fully explain the absence of consistent shortcutting behaviour in later studies.

Generally, the animals used in the Sunburst maze experiments spanned very similar age ranges: The youngest were approximately 2 months (Harley 1979 acquisition group), most were around the age of Tolman et al.'s (1946) animals: 3–4 months (Tolman et al. 1946; Gentry et al. 1947, 1948; Kendler and Gasser 1948; Ritchie 1948; Kendler and Mencher 1949; Dawson 1972; Michik 1973; Muir and Taube 2004 personal communication), with the oldest around 6–8 months (Birch and Korn 1958; Harley 1979 retention group) (Table S1). However, Tolman et al.'s (1946) experimental group was slightly unusual in that it was made up exclusively of female rats. While some replication studies have similarly used females (Birch and Korn 1958; Muir and Taube 2004), most have instead used males (Chamberlain 1947; Kendler and Gasser 1948; Ritchie 1948; Kendler and Mencher 1949; Harley 1979) or did not specify the sex (Gentry et al. 1947; Young et al. 1967) (Table S1). This difference is noteworthy because male and female rats have been found to use different cues when solving spatial tasks (Williams et al. 1990; Roof and Stein 1999). More specifically, males tend to use geometric information (such as the shape of a maze), while females rely more on visual landmarks (Rodríguez et al. 2010). Given that Tolman et al. (1946) provided a distinctive landmark near to their goal location, one possibility is that their female subjects were more likely to beacon towards this landmark and take the shortcut. However, Muir and Taube (2004) and Birch and Korn (1958) did not observe shortcutting in female rats in very similar mazes. Furthermore, both Dawson (1972) and Michik (1973) compared male and female rats in the Sunburst task and found that males actually chose paths closer to the shortcut while females, on average, selected paths adjacent to the training route. Similarly, in humans, male participants consistently outperform female participants in place navigation tasks (Munion et al. 2019; Nazareth et al. 2019) and are more likely to use shortcutting (Boone et al. 2018, 2019); because of this, we may expect that subsequent replications using male subjects would be more likely to find evidence of shortcutting, not less. Overall, it is unclear if male and female rats exhibit differences in their likelihood to use shortcuts, but given all of the evidence together, it seems unlikely that the sex of the subjects could explain the difficulties in replicating Tolman et al.'s (1946) findings.

### 3.3 Training and Pretraining

An often overlooked, and unique, aspect of Tolman et al.'s (1946) rats, is that they were pretrained in the Tryon maze described above (Tryon 1940; Munn 1951; Tolman et al. 1929). Over 18 days of progressive training, rats had to find the maze's exit through a succession of T‐mazes (see Figure S1 for schematic and full protocol), which likely relied on learning an egocentric sequence of actions (Arleo and Rondi‐Reig 2007). This could have encouraged rats to use a similar strategy in the Sunburst maze, biasing animals towards repeating learned action sequences. Tolman et al.'s (1946, 18) data support this: During the Sunburst test, every rat first attempted to run up the original training alley. However, it is unclear how this history of sequential response learning could have led rats to choose the shortcut; more plausibly, the pretraining may have indirectly made the rats ‘maze‐wise’—highly habituated to testing procedures, and more comfortable exploring the apparatus than the largely naïve animals used in later replications. While some replication studies also employed pretraining in a separate maze (Gentry et al. 1947; Experiment I; Birch and Korn 1958; Dawson 1972; Michik 1973), most did not (Ritchie 1946, 1948; Gentry et al. 1947 Experiment II–V, Gentry et al. 1948; Kendler and Gasser 1948; Kendler and Mencher 1949; Muir and Taube 2004) or did not specify if pretraining was used (Young et al. 1967; Harley 1979) (Table S1). This variation in pretraining procedures complicates comparisons across studies and raises the possibility that Tolman et al.'s (1946) original findings reflect, at least in part, the behavioural predispositions created by their extensive pretraining regimen.

The Sunburst experiment itself also had a 5‐day training period (Figure 1c, left), during which rats were gradually introduced to the maze in its training configuration (Tolman et al. 1946). The number of trials and days involved in this training vary across studies, but this does not seem to fully explain the variability in results either. In more details, over the first 3 days, animals were placed successively farther from the reward along the outbound path. On Days 3 and 4, they completed five full runs from the start box to the reward, and on Day 5, they were given the Sunburst test (Figure 1C, right). Subsequent replications using variants of the original maze design adopted comparable but often more intensive training protocols. Ritchie (1948) followed a similar progressive schedule but gave his rats a larger total number of full‐path trials (20). Gentry et al. (1948) trained their animals one trial per day for 11 days. Harley (1979) provided eight trials per day for 6 days (48 total), plus two additional trials on the test day. Dawson (1972) gave his rats seven trials from the start to the reward across 3 days. Kendler and Gasser (1948) and Kendler and Mencher (1949) varied training intensity across groups, giving four trials per day until animals had achieved 0, 5, 20 or 100 correct runs. Across these studies, rats typically received far more training than in the original design, raising the possibility that these animals were overtrained and may have shifted from place‐based to response‐based navigation (Ritchie et al. 1950; Packard and McGaugh 1996), which could contribute to the consistent absence of shortcutting. However, both Birch and Korn (1958) and Gentry et al. (1947) closely reproduced the original apparatus and followed the progressive training schedule verbatim, yet neither study found evidence of shortcutting. Human virtual reality replications by Wilson and Wilson (2018) and Doner et al. (2023) gave their participants a similar number of training trials to the original study, over the course of a single session, yet, both studies concluded that participants' choices were guided primarily by the light cue rather than spatial inference. Together, these findings indicate that differences in training procedure likely cannot account for the failure of later studies to reproduce Tolman's shortcutting effect.

### 3.4 Lights and Geometry

There are several concerns with the physical design of Tolman et al.'s (1946) Sunburst maze. The first is the placement of a light directly behind the reward location. This raises the possibility that rats simply approached the light stimulus rather than inferring the reward's spatial position, a navigational strategy known as ‘beaconing’ or stimulus‐approach (Arleo and Rondi‐Reig 2007). This concern has been raised repeatedly in subsequent studies (Wilcoxon and Waters 1948; Hilgard and Marquis 1961, 224; O'Keefe and Nadel 1978, 71; McDonald and Pellegrino 1993, 50; Thinus‐Blanc 1996, 10; Wiener and Taube 2005, 232; Shettleworth 2009, 299; Foreman and Gillett 2013, 88; Warren 2019; Buatois and Gerlai 2020; Bouchekioua et al. 2021; Miller 2021; Horner et al. 2025) and is discussed in more detail later (Section 4). While map‐based navigation requires the animal to use an internal representation of space to compute a route towards a goal that is not necessarily directly visible, beaconing involves a straightforward attraction to a salient cue (Arleo and Rondi‐Reig 2007). Furthermore, Tolman et al. (1946) chose to make this light the only source of illumination in the room. When a light source is the only provided extramaze cue, rats will often strongly base their behaviour around it, to the point of running off the edge of a maze when the light is moved (Scharlock 1955). This strict cue‐centric type of navigation is antithetical to the map‐based learning Tolman et al. (1946) aimed to test.

Finally, the maze had no side‐walls except along the initial segment of the training path. While apparently not an issue for Tolman et al.'s (1946) experiment, this feature may have introduced biases in subsequent studies for two reasons: (1) Many small animals exhibit thigmotaxis, a preference for locations next to walls (Treit and Fundytus 1988); and (2) combined with the single light source, these walls would have cast shadows across some arms and many small animals exhibit phototaxis, a preference for darker areas (Crawley 1985).

### 3.5 Uncontrolled Cues

In addition to visual cues, uncontrolled olfactory cues may also have influenced behaviour in Tolman et al.'s (1946) experiment. During testing, only the central platform was rotated, leaving the choice arms in place, allowing rats to potentially follow self‐deposited scent trails, such as urine, from previous animals. Several replications used similar methods (Gentry et al. 1947; Kendler and Gasser 1948; Kendler and Mencher 1949; Birch and Korn 1958), but did not observe shortcutting, suggesting that odours alone are unlikely to account for Tolman et al.'s (1946) findings. Studies that controlled for odour cues more thoroughly, by rotating both the platform and the paths (Dawson 1972; Michik 1973) or dismantling and washing the maze daily (Gentry et al. 1948) did not report evidence of shortcutting either. The same absence of shortcutting was observed in studies that did not report any odour‐control procedures (Ritchie 1948; Young et al. 1967; Muir and Taube 2004). Taken together, these findings suggest that while odour cues were rarely adequately controlled, they are unlikely to explain the apparent shortcutting observed in the original study.

A further possibility is that the behaviour observed by Tolman et al. (1946) was influenced by another type of unreported environmental cue within the experimental room. Ritchie (1947), working in Tolman's laboratory just a year later, discovered that rats in a plus maze consistently oriented their choices towards the location of their home cages, which were stored in a fixed position within the testing room. When the home cages were removed, the change in performance of the animals ‘was astounding’ (p. 32), demonstrating that such cues can exert a strong directional influence. Restle (1957) later highlighted home cages and cage racks as particularly salient orienting stimuli for rats. Tolman et al. (1946) did not describe the spatial arrangement of their experimental room nor mention the presence of home cages, cage racks, windows or doors, but these features were likely present. Having rat cages in experimental rooms seems to have been common at the time (Ritchie 1948; Birch and Korn 1958) and Tolman (1948) implies that Ritchie's (1948) later maze experiments were conducted in the same room and apparatus as the original study (‘the rats were again run across the table’, p. 204). If a similar arrangement existed during the Sunburst test, auditory or olfactory cues from conspecifics could have drawn animals preferentially in that direction. While speculative, this possibility underscores the potential impact of unreported environmental asymmetries on navigation behaviour in early maze studies.

### 3.6 Summary

Taken together, each of these methodological and design choices may have introduced strong biases in how the rats behaved during the test phase. The lenient scoring rules and post hoc exclusions could have inflated the rate of choices directed towards the reward location, while the use of selectively bred and pretrained rats may have favoured response patterns that generalized poorly across other studies. Additionally, the combination of a single light source and the partial walling of the maze created pronounced visual asymmetries across different paths. Some would have been more brightly illuminated, while others were partly in shadow, and the availability of wall‐adjacent regions varied between them. Although it is not possible to estimate how these asymmetries affected the rats' choices, these uncontrolled factors could have systematically influenced exploratory patterns. It is important to note that no single factor satisfactorily explains the discrepancy between the original and subsequent studies. The key issue is therefore not that these features necessarily favoured the shortcut arm, but that they created uneven sensory conditions across the maze, making it difficult to attribute any directional preference unambiguously to spatial inference rather than to the structure of the apparatus. Thus, what has often been interpreted as compelling evidence of cognitive maps may, on closer inspection, owe much to the peculiarities of Tolman et al.'s (1946) apparatus, subjects and scoring conventions. These methodological observations are not without precedent; Kendler (1952, 270) commented that ‘Tolman has violated methodological requirements which he himself promulgated’, and similar concerns have been raised regarding Tolman and Honzik's (1930) detour task (Ciancia 1991).

## 4 How Do Subjects Behave in the Sunburst Maze?

**FIGURE 3:**
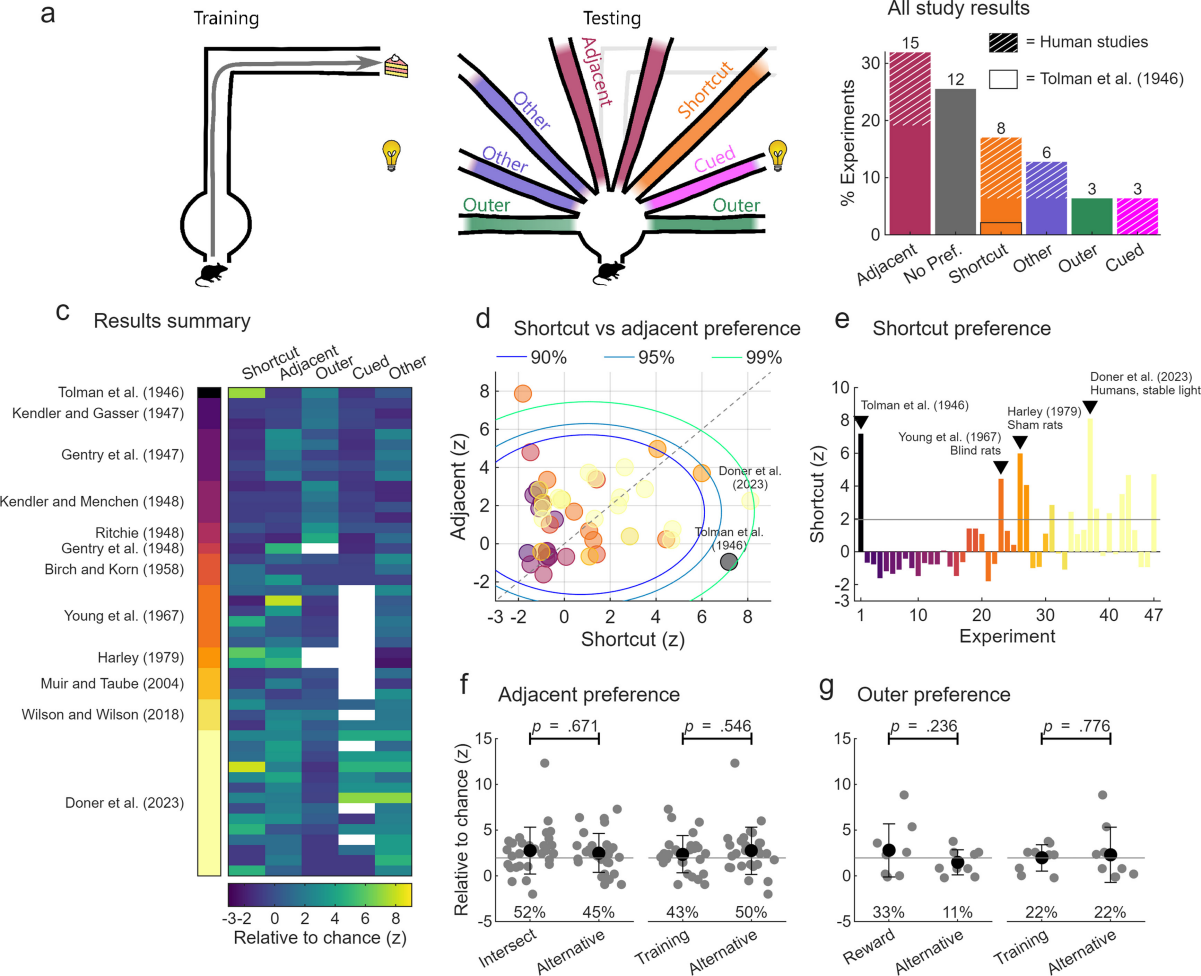
The prevalence of shortcutting across studies has been very low. (a) Schematized example of shortcut training (left) and testing (right) with the possible categories of path choices labelled. (b–e) See Methods: *Experiment categorization*. (b) Experiments were categorized based on the path type chosen most frequently, among paths preferred at an above chance level. If no path was chosen at an above chance level the result was categorized as ‘no pref.’ (no preference). (c) Across all replications, preference for these paths relative to chance. (d) The preference for shortcut vs. adjacent routes across studies, also shown are the 90%, 95% and 99% confidence intervals computed from the distribution of experiments. Colours correspond to vertical colorbar in (b). (e) Preference for the shortcut route across studies. Colours correspond to vertical colorbar in (c). Horizontal line denotes significance at the *p* < 0.05 level. (f) Left: preference, relative to chance, for the adjacent path which is more likely to intersect the training route (‘intersect’) or the other adjacent path (‘alternative’). Only experiments where at least one of the adjacent paths was chosen at an above chance level are shown. Horizontal line denotes significance at the *p* < 0.05 level, text gives the percentage of experiments in which each path was chosen above chance. No preference was observed. Right: same but for the adjacent path which heads in the same direction as the first turn of the training route (‘training’) or the other adjacent path (‘alternative’). (g) Left: same as f but for the outer path on the rewarded side of the maze (‘reward’) or the other outer path (‘alternative’). Right: same but for the outer path, which heads in the same direction as the first turn of the training route (‘training’) or the other outer path (‘alternative’).

Following Tolman et al.'s (1946) report, numerous research groups sought to replicate their shortcutting findings. Including the original study, these comprise 13 studies reporting 47 separate experiments to date (see Methods: *experiment categorization*). Shortcutting behaviour has exceeded chance in only 17% of these (Figure 3b–e ‘shortcut’). For comparison, 13% of experiments found an above‐chance preference for paths with no remarkable features (Figure 3b ‘other’). The two most common findings across these studies are that subjects seemingly choose paths without exhibiting a specific preference (26%; Figure 3b ‘no pref.’) or choose a path immediately adjacent to the training path (32% of experiments; Figure 3b ‘adjacent’). The remainder of studies typically fall into one of two categories: (1) subjects approach or avoid visual cues such as light sources (6%; Figure 3b ‘cued’); or (2) they select one of the outermost paths (6%; Figure 3b ‘outer’).

Compared to other studies, subjects in Tolman et al.'s (1946) experiment were extremely biased towards the shortcut and rarely chose the two paths adjacent to the training route, a pattern that clearly stands apart (Figure 3d). None of the six studies published in the 20 years following Tolman et al. (1946) replicated the shortcutting preference (Figure 3e). The first experiment that found a significant shortcut preference was in a subset of four blind rats (Young et al. 1967) with only three more groups finding some degree of shortcut preference subsequent to this (Dawson 1972 possibly in male rats; Harley 1979 in one experiment of two; Doner et al. 2023 in humans); see also Michik (1973 male rats) (Figure 3e). Overall, significant shortcutting, especially to the degree found by Tolman et al. (1946) and with its absence of adjacent path preference, is a clear outlier in comparison to the literature (Figure 3d). This points to a very different question: If they are not using a cognitive map, how are subjects really solving the Sunburst maze?

### 4.1 Preference for the Adjacent (Central) Paths

Tolman et al. (1946, 20) proposed that animals selected the shortcut path because they had learned the spatial location of the reward, formed an expectation of reward at that location and chose the shortcut as it provided the most direct route to the goal. However, Olton (1979, 589), critical of these results, argued that because training involves a circuitous outbound path, a rational response when this is blocked is to attempt to rejoin the training path beyond the obstruction, rather than try to infer a novel direct route. Indeed, in experiments where animals must navigate around obstructions, they overwhelmingly take paths which circumscribe the obstacle, staying adjacent to it until a familiar path is found or the goal can be approached directly (Gengerelli 1930; Collett et al. 2001; Kimchi and Terkel 2004; Shamash et al. 2021).

Consistent with this idea, a prominent behavioural pattern observed across numerous experiments is that when the original training path is blocked, subjects often chose one of the paths immediately adjacent to it (32% of experiments; Figure 3a,b) (Gentry et al. 1947, 1948; Birch and Korn 1958; Young et al. 1967; Harley 1979); see also the female rats of Dawson (1972) and Michik (1973). This effect persists even when the training and adjacent paths head away from the reward location (Gentry et al. 1948) but is disrupted when the start orientation is changed between training and testing (Ritchie 1948; see Section 4.5). Given multiple choice trials, rats will switch from one adjacent path to the other, suggesting that it is the adjacency to the training path that is preferred rather than the path's heading direction (Harley 1979). Harley (1979, 289) observed that, in addition to her published results, 126 rats tested in her circular sunburst maze chose adjacent arms 90% of the time, confirming that this is a prevalent and reproducible effect.

In addition to choosing adjacent arms even if they head away from the reward location, rats exhibit the same adjacency effect in Sunburst maze designs that incorporate a fully circular, rather than semicircular, choice area (Gentry et al. 1948; Harley 1979). However, we note that in one of Harley's (1979) experiments, the rats did prefer the shortcut (also adjacent) path over the other adjacent path. This was not replicated in a 2nd experiment where a second group of control rats preferred both adjacent paths equally (discussed more in Section 4.3). A general preference for adjacent paths occurs in enclosed mazes where no visual cues are available (Young et al. 1967) and in transparent mazes where it should be clear that the adjacent path does not head towards the reward location (Harley 1979). Rats will continue to choose adjacent paths even if the training route is removed during the test phase, and if the adjacent paths themselves are removed rats show a preference for the next most adjacent ones instead (Young et al. 1967). These results suggest that the preference for these adjacent paths is not based on a sensory feature, or physical aspect of the paths themselves, but rather the fact that they are physically adjacent to the training path.

However, to complicate this conclusion, untrained rats that have never experienced the training route also exhibit an adjacency tendency, which is no longer observed if the training route is physically removed (Gentry et al. 1947). This suggests that the preference for adjacent paths may result, at least initially, from the physical structure of the maze. The fact that trained rats continue to exhibit an adjacency preference under the same circumstances (Young et al. 1967) suggests that while naïve animals exhibit a preference for adjacent paths based on physical aspects of the maze, this preference may become strengthened during training, at which point it persists regardless of physical changes to the apparatus.

Why might naïve animals be attracted to the adjacent paths? As described above, in some Sunburst maze designs the adjacent arms have a wall on the training path side, or are in shadow, which may attract safety‐seeking animals (Crawley 1985; Treit and Fundytus 1988). More generally, subjects are often drawn towards central options, for instance, recalling a stimulus as being closer to the centre of a category (Huttenlocher et al. 1991), perceiving items in the centre of an array as preferable (Valenzuela and Raghubir 2009) or fixating on central visual cues more (Tatler and Vincent 2009). Given the semicircular design of most Sunburst mazes, the adjacent and training paths are also the most central ones, which may lead to their overall preference when presented as an array. In circular maze designs (Gentry et al. 1948; Harley 1979) the training path may define the ‘center’ of the array, and this could be tested by looking at the responses of untrained animals.

Why does training strengthen the adjacency effect? One possibility is that the responses repeatedly made on the training path increase a bias for adjacent paths. Indeed, some studies have found that altering the length or complexity of the training path influences the likelihood of an adjacency bias (Ritchie 1946, 108 preliminary Experiment 3; Young et al. 1967) suggesting that the sequence of self‐motion cues learned on the training path plays a role. However, Gentry et al. (1947; Experiment IV) found no effect of path modifications on adjacent path choices; this experiment was based on the largest number of subjects and most closely replicated the original Sunburst study. Consistent with these findings, human subjects also exhibit the adjacency effect in virtual reality environments (with or without proprioceptive feedback) (Doner et al. 2023). Testing across all experiments we found that the direction of the first turn along the training path had no significant effect on the preference for either adjacent path (Figure 3f right). Thus, it seems unlikely that the sequence of responses made on the training path influences the animal's preference for adjacent paths.

Another possibility is that during training, subjects learn a stimulus‐triggered response (Arleo and Rondi‐Reig 2007), otherwise known as a place recognition‐triggered response (Trullier et al. 1997). Here, the spatial context (e.g., visual landmarks or geometry), instead of a single cue, acts as a stimulus to trigger a specific motor response (or first‐order vector: Bouchekioua et al. 2021). For example, animals in the Sunburst task may learn during training to move northwards from the choice platform; in the test phase, the paths allowing the most similar response are the ones adjacent to the initial training route. This strategy is not consistent with shortcutting or a cognitive map hypothesis as the animal does not need to have an internal representation of the relations between its current location and other places in the environment (Trullier et al. 1997). In this view, adjacency preference poses a serious challenge to the claim that animals and humans solve the Sunburst task using a cognitive map.

In most Sunburst maze designs, one of the adjacent paths is more likely to intersect the long segment of the training path. Coming back to Olton's (1979) prediction that subjects should attempt to rejoin the training path beyond the obstruction, do animals choose this intersecting path more than the alternative? We tested this across all experiments and found that this was not the case (Figure 3f left). This further supports the conclusion that many animals in these tasks are instead simply repeating a stimulus‐triggered response that was reinforced during training rather than using a spatial map.

### 4.2 Absence of Preference

Surprisingly, one of the most common results from the set of Sunburst maze studies is the absence of specific path preference (26% of experiments; Figure 3e ‘no pref.’). The fact that the test environment was novel might explain this: Indeed, with the exception of Harley (1979) for which the Sunburst arms were visible but blocked throughout training, in all other paradigms, the new arms were added at the time of testing. Rodents often respond to novelty with anxiety (Ennaceur et al. 2006) or exploration (Whiting and Mowrer 1943) and in both cases may exhibit little interest in reward. Novelty‐driven exploration might lead to path choices that are unrelated to goal‐directed navigation. It is also possible that the subjects were lost in the new environment (discussed in Section 5.2); indeed, hippocampal place cells, which are now believed to underlie the cognitive map, either fully or partially ‘remap’ when the geometry of an environment is changed (Muller and Kubie 1987; Lever et al. 2002; Grieves et al. 2016). Overall, while it is unclear why this absence of preference occurs, it is one of the most common findings and should not be ignored.

In addition to this absence of preference in many of the studies, within each experiment, a subset of subjects often avoided choosing a path at all. This made up only a small proportion of the rats in the original study (5.4% or 3/56, Tolman et al. (1946)), but it was more prominent in some replication attempts, for example, Birch and Korn (1958) where 53% of rats made no final choice in Experiment I (Table S1). Woodworth (1971, 632) noted that, in the Sunburst test, the training path and reward box are visibly absent, so rats should be confused as to where to go and not select a path at all, rather than shortcutting to a location that is visibly unrewarded. Another possible explanation is that, at least in the experiments of Kendler and Gasser (1948), Kendler and Mencher (1949) and Birch and Korn (1958), the rats' actions in the test phase were primarily driven by anxiety. This conclusion is supported by the fact that rats exhibited anxiety‐related behaviour in Kendler and Gasser (1948) and Kendler and Mencher (1949) task (Chamberlain 1947, 48 notes that rats urinated and defecated significantly more during the test phase).

This pattern of results highlights a common issue with shortcutting studies: on the one hand, use of a shortcut is only supportive of the cognitive map hypothesis if it is novel, i.e., either the animal makes a novel spatial inference or combines previously‐experienced paths in a new sequence (Bennett 1996; Grieves and Dudchenko 2013). On the other hand, testing in a novel environment usually triggers a switch from exploitation to exploration or even novelty‐induced anxiety, which can interfere with testing extant spatial knowledge. Future tests of shortcutting should account for these aspects in order to provide reliable results.

### 4.3 Choosing the Shortcut

Across all available studies, including the original, only a small fraction report a significant preference for the shortcut path in the Sunburst maze (~17%). Tolman (1948) interpreted these findings as compelling evidence that rats formed a cognitive map of their environment: They learned to expect food at a specific place during training, and in the Sunburst test took the path which took them directly to this location (Tolman et al. 1946). However, a number of simpler mechanisms can result in shortcut‐like behaviour (Bennett 1996; Gibson 2001).

One long‐standing concern is that shortcutting may arise from beaconing, where animals approach a conspicuous cue near the reward rather than computing its spatial location. For example, in an influential study, Gould (1986) reported that honeybees took novel shortcuts between two foraging sites. However, in subsequent experiments, several studies were unable to replicate Gould's (1986) findings unless prominent landmarks at one foraging site were visible from the other (Menzel et al. 1990; Wehner and Menzel 1990; Wehner et al. 1990), undermining the novel shortcutting claim. Tolman et al.'s (1946) maze contained a bright, directional light placed directly behind the reward. Because this light was contained within a reflector, it is unclear how visible the light would have been from the shortcut path, or which path would have matched the training alley most closely. However, most rodent replications that included a comparable light did not observe convincing shortcutting (Gentry et al. 1947, 1948; Birch and Korn 1958), indicating that beaconing alone is an insufficient explanation, though a possible contributing factor. Human studies provide a mixed picture: in one of their experimental groups, Wilson and Wilson (2018) found above‐chance shortcutting that did not appear to reflect cue‐approach, whereas Doner et al. (2023) observed strong beaconing to the light that persisted even when it changed position, consistent with evidence from other human shortcutting studies (Foo et al. 2005). Beaconing and the significance of the light cue are discussed further in Section 4.4.

A second explanation is vector integration, where animals accumulate a sequence of self‐motion vectors during training and sum them to generate a direct egocentric vector to the goal at test (Etienne et al. 1998; Redish 1999, 18; Kubie and Fenton 2009; Ekstrom et al. 2014). Consistent with this, Young et al. (1967) found considerable shortcutting in blind rats, i.e., animals unable to rely on distal visual cues, but fully capable of using self‐motion–based updating. Computational models based on vector summation likewise reproduce shortcutting in Sunburst‐like mazes (Banino et al. 2018; Edvardsen et al. 2020; Bouchekioua et al. 2021). However, because these vectors are based on estimations and are open to errors (Maurer and Séguinot 1995), these models typically produce graded distributions, with increased selection of the shortcut as well as its neighbouring alleys. By contrast, Tolman et al.'s (1946) rats showed an unusually strong preference for the shortcut and *subchance* selection of neighbouring alleys, a pattern that is difficult to reconcile with noisy path integration mechanisms.

Across all of the Sunburst studies, the conditions under which shortcutting has been replicated are limited and inconsistent. Harley (1979) found shortcutting in one experiment using a simplified Sunburst maze, but in her design, the shortcut coincided with an adjacent path, and a second experiment in the same study did not reproduce the shortcut preference (discussed further in Section 5.2). Dawson (1972) and Michik (1973) reported that male rats tended to choose alleys closer, on average, to the shortcut, but their analyses were based only on mean alley numbers, obscuring the underlying choice patterns. Young et al.'s (1967) blind rats preferred the shortcut but represented a small number of animals (*N* = 4), and methodological details, particularly about the order and frequency of choices, were insufficient to determine whether genuine inference occurred. Although finding some evidence of shortcutting, studies using humans concluded that the subjects strongly based their behaviour on the position of a visual cue (Wilson and Wilson 2018; Doner et al. 2023). Outside the Sunburst literature, several species, including dogs (Chapuis and Varlet 1987) and Chimpanzees (Normand and Boesch 2009), have been reported to shortcut (see also detouring of jumping spiders in Jackson and Wilcox 1993), but these findings often admit alternative explanations or have proven difficult to replicate. By contrast, a substantial body of work indicates that animals generally do not take truly novel shortcuts without prior exposure to those routes (Maier 1932; Erhart and Overdorff 2008; Grieves and Dudchenko 2013; Porter and Garber 2013).

Viewed in this broader context, Tolman et al.'s (1946) findings appear highly unusual. No subsequent study has reproduced the strong and specific shortcut preference they reported, and most experiments across rodents, humans and other species find limited or inconsistent evidence for spontaneous shortcutting under equivalent conditions. Whether Tolman et al.'s (1946) animals relied on a unique combination of cues, an idiosyncratic environmental asymmetry or some other uncontrolled factor remains unresolved, but the pattern of results across the literature strongly suggests that the Sunburst maze is not a reliable assay of map‐based spatial inference.

### 4.4 Inconsistent Influence of the Light

Are rats in Tolman et al. (1946) using a cognitive map to select the shortcut to the goal, or are they simply beaconing towards the light source? This critique has been raised repeatedly in subsequent reviews (Wilcoxon and Waters 1948; Hilgard and Marquis 1961, 224; O'Keefe and Nadel 1978, 71; McDonald and Pellegrino 1993, 50; Thinus‐Blanc 1996, 10; Wiener and Taube 2005, 232; Shettleworth 2009, 299; Foreman and Gillett 2013, 88; Warren 2019; Buatois and Gerlai 2020; Bouchekioua et al. 2021; Miller 2021; Horner et al. 2025). O'Keefe and Nadel (1978), for example, noted that ‘Unfortunately, a light H was located at the goal, making a strong place‐learning interpretation of these data impossible’ (p. 71). Tolman et al. (1946, 21) seemed aware of this limitation and argued that (1) the light appeared straight on in the last segment of the training path, while this direct view of the light was not available from any path in the test apparatus (because the lamp had a reflector oriented towards the training path). However, in the test phase, rats were allowed to ‘explore’ different paths before making a final choice and could have selected the one judged to match the training view most closely; (2) if the rats used the light as a beacon, we would expect them to choose other paths facing the light source with similar frequency (such as #5 and #7; Figure 1c), which they did not. However, in the test phase it is unclear if the other paths would have indeed presented a light source that was directly ahead (i.e., arm #7) or as bright (i.e., arm #5) as on the training path, undermining this prediction; (3) the light source was a spatial landmark and such cues are necessary for an animal to associate reward with a specific location. This is true, but it is undermined by the fact that the maze was in a darkened room and there were no other visual cues explicitly available. From a contemporary perspective, it is surprising that the reward location was chosen to coincide with a salient cue, instead of being in an uncued but spatially stable location, with its position determined with respect to multiple distal cues (Morris 1981). Ultimately, the concern is that subjects may treat the light as an indicator of the reward location, without relying on a cognitive map of the environment to reach the reward. Interestingly, a number of subsequent studies investigated this possibility.

For example, Birch and Korn (1958) and Gentry et al. (1947) closely reproduced Tolman et al.'s (1946) apparatus and procedures with the aim to thoroughly test the influence of the light source, which they predicted acted as a simple stimulus guiding the animal's behaviour (Birch and Bitterman 1949). In these experiments, rats were trained in the Sunburst maze with a light source either immediately above the reward location or symmetrically placed on the opposite side of the maze. During the test phase, the light source was moved to different positions around the maze (Figure 2a and Figure S2). Across all of these configurations, rats did not choose the paths leading towards the light source(s) more than chance. Dawson (1972) similarly reproduced Tolman et al.'s (1946) apparatus and procedures, including the light source, but did not observe a strong preference for the shortcut path. Perhaps most convincingly, Gentry et al. (1948) trained rats in a circular version of the Sunburst maze. The reward location and a light source were placed at one side of the central choice platform, while the training path initially headed away from this platform on the opposite side (Figure 2g and Figure S8). In the test phase, one path led directly to the reward location and light source, but the majority of rats chose paths adjacent to the training route and directly *away* from the light source. A number of additional experiments have also reported animals either ignoring light sources or choosing paths heading away from them (Ritchie 1946; 1948; Chamberlain 1947; Kendler and Gasser 1948; Kendler and Mencher 1949), although in the case of Ritchie (1948) rats were likely using an illuminated wall as a beacon (Ritchie 1946, 107: see Experiment III, preliminary test 1). Together, these results strongly suggest that rats did not simply approach the light source in Tolman et al.'s (1946) experiment. Intriguingly, these results are not necessarily consistent with those of human participants.

For example, Wilson and Wilson (2018) and Doner et al. (2023) reproduced Tolman et al.'s (1946) design using three‐dimensional virtual environments. Groups of participants were trained in these mazes with a light source immediately adjacent to and above the reward location, which was then moved to different locations around the maze during the test phase (Figure 2c and Figure S4). Both studies found that the choice of path in the test phase was significantly influenced by the position of the light source. When the light was moved in the test phase, the number of participants choosing the correct shortcut route dropped, and many instead chose the route heading directly towards the light, even if this took them away from the reward location. Providing extramaze cues, or vestibular and kinaesthetic feedback, did not greatly change these results (Doner et al. 2023). Together, these studies suggest that in human participants, unlike rodents, path choices in the Sunburst test are significantly influenced by the location of a light source. These results may reflect the increased dependence on visual cues by navigating humans (Foo et al. 2005; Ekstrom 2015), but it is also important to point out that, unlike the platform‐based design of Tolman et al.'s (1946) maze, every maze segment in these virtual environments had tall (4.4 m high) side‐walls that prevented viewing the rest of the maze. It is unknown if removing these walls, so that it is clear to the participants that the reward and training route are not present, would lead to different results.

In summary, although often cited as the main flaw in Tolman et al.'s (1946) experimental design, evidence suggests that the light source did not function as a beacon to the reward. Across the many replication studies reviewed, animals almost never chose paths leading towards a light; such choices occurred more than chance in only 6% of experiments, and exclusively with human participants. The real issue is that relying on a single light deprived animals of the multiple distal cues normally required for map‐based navigation, thereby reducing the likelihood they would use such a strategy. Moreover, without dissociating the light from the reward location—a control that Tolman et al. (1946) did not implement—or more clearly describing which of the test paths provided a direct view of the light, it is impossible to rule out beaconing.

### 4.5 Preference for the Outermost Arms

The most chosen path in Tolman et al.'s (1946) experiment was the shortcut route, but the second most frequently chosen path was the outermost (reward‐side oriented) one (#1) with 9/53 (17%) of rats choosing this path (Figure 1d). This result was somewhat overlooked by Tolman et al. (1946) who suggested that it was an ‘artifact of the experimental apparatus’ (p. 18) and conclude their study with the assertion that 36 percent of the rats chose the shortcut route and the other routes were chosen ‘in a chance fashion’ (p. 24), even though this outermost arm was chosen at a rate twice that expected by chance (8.3%). As discussed earlier, the six paths on the left side of Tolman et al.'s (1946) maze were not included in the final analysis and rats choosing those paths were allowed to make a second choice. This means that we do not know how many rats chose the opposite outermost path (#18). To complicate the issue, Tolman et al. (1946) note that four of these second‐choice rats switched their choice to Path #1 (excluding the choices of these rats still results in an above chance rate for Path #1 at 5/53 or 9.4%). Interestingly, in 6% of the experiments reviewed, the significantly preferred choice of path in the Sunburst test was also an outermost one (Figure 3b).

For example, Ritchie (1946, 1948) tested rats in a version of the Sunburst maze where the start position during testing was opposite to the start position used during training (Figure 2e and Figure S6). Ritchie (1946) predicted that rats would navigate to the reward location, regardless of the change in start position, reinforcing Tolman et al.'s (1946) shortcut findings. Instead, 57% (24/42) of the rats chose the outermost path at a 90° angle to the start of the training route, on the side of the reward (i.e., #10 or #1, depending on reward location, see Figure S6). Similarly, Kendler and Gasser (1948) and Kendler and Mencher (1949) tested rats in a simplified, T‐maze‐like variant of the Sunburst maze (Figure 2f and Figure S7) (see also Chamberlain 1947 unpublished thesis). While very few animals chose the shortcut path leading to the reward location, many exhibited a strong preference for the outermost paths at a 90° angle to the start of the training route. With additional testing, rats demonstrated a further preference for the outermost path heading towards the rewarded side of the room. Consistent with this training effect, Birch and Korn (1958) also found that, with increased training, rats chose the outermost arms of a Sunburst maze more frequently in test trials (Figure S2f, g).

Why does this preference for the outermost paths occur? We propose three explanations. The first relies on the tendency of rodents to first explore the periphery of new environments (‘thigmotaxis’; Treit and Fundytus 1988; Fonio et al. 2009). Since the Sunburst maze used during the test was completely new to the animals, they may have been inclined to explore along the boundaries of the maze, which would include the outermost arms.

The second explanation is that, during training, animals learn to use a route‐based (sequential egocentric) strategy: memorizing a sequence of egocentric motion directions (Rondi‐Reig et al. 2006; Arleo and Rondi‐Reig 2007). During testing, when the training path is blocked, they then attempt to execute the next response in the sequence, which in the original design is turning 90° left followed by two 90° right turns. In the test phase, the paths most closely aligning to these responses are the outermost ones. Consistent with this view, animals typically exhibit a preference for the path corresponding to the first training response, which often does not head towards the reward (Gentry et al. 1947; Kendler and Gasser 1948; Birch and Korn 1958; Wilson and Wilson 2018). However, this is not always the case (Tolman et al. 1946; Ritchie 1948) and when tested across all studies we found no significant bias for the outermost path that corresponds to the first response made on the training route or the outermost path heading in the direction of the reward (Figure 3g), undermining this route‐based explanation.

A last possibility is that, at least in some cases, sensory features seem to play an important role in this bias for outermost paths. For example, Kendler and Gasser (1948) concluded that untrained rats exhibited a preference for outermost paths in their maze because they were simply the darkest. This conclusion was strongly supported by the finding that if the light source was moved around the maze, rats consistently chose the outermost path heading away from it (Chamberlain 1947; Experiment II). Ritchie (1946) similarly found that his rats were biased by the position of an illuminated wall in the room, and when this cue was omitted rats ‘showed no preference for any one path’ (Experiment III, preliminary test 1, p. 107). Overall, these findings suggest that in many cases, outermost path preferences may simply be an artifact of the experimental apparatus as Tolman et al. (1946) originally suggested.

## 5 What Does the Sunburst Maze Measure?

**FIGURE 4:**
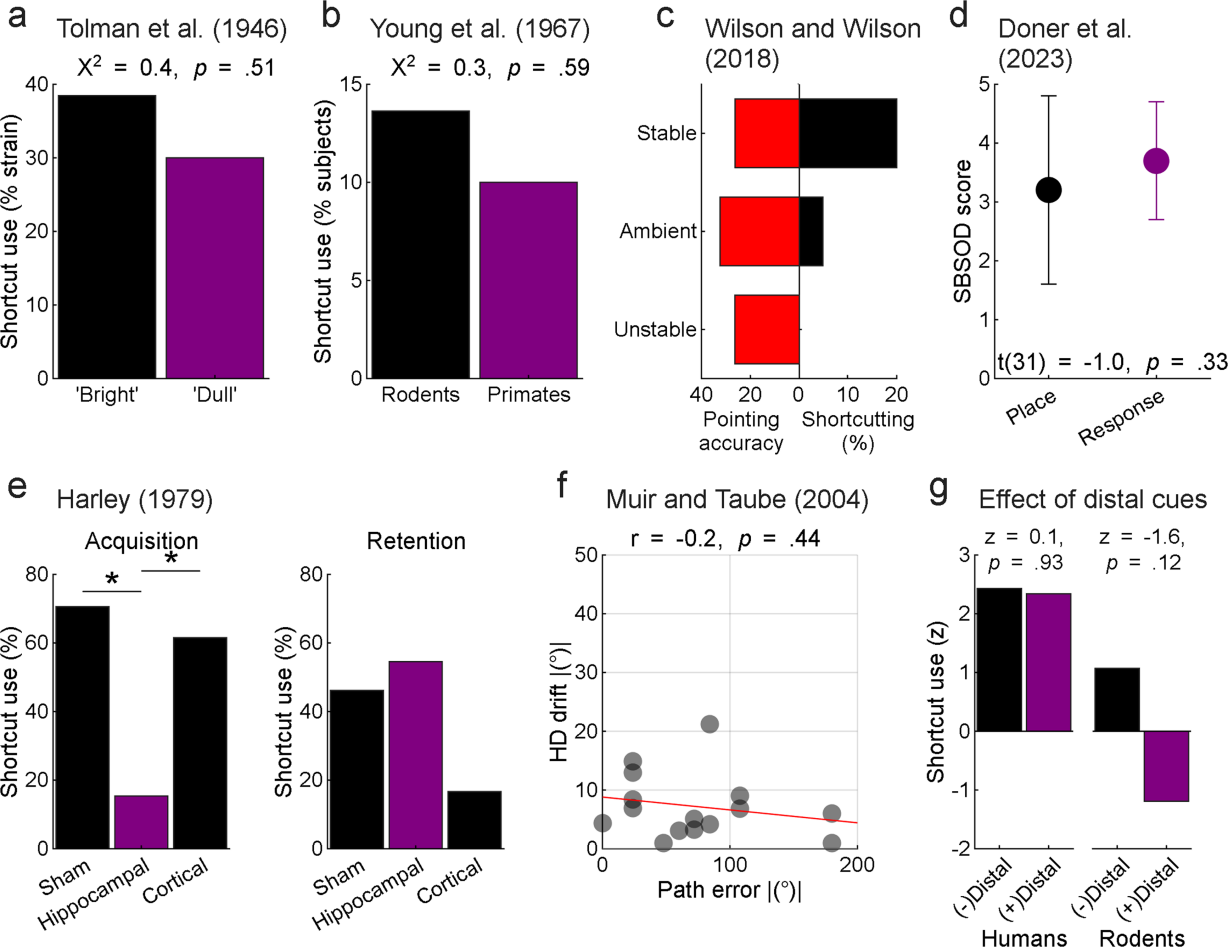
Sunburst performance is not related to other measures of spatial knowledge. (a) ‘Bright’ rats selectively bred to perform well in a complex maze task did not choose the shortcut significantly more than ‘dull’ rats bred to perform poorly (Tolman et al. 1946). (b) Monkeys ( *Saimiri sciureus* ) do not choose the shortcut significantly more than rodents (Young et al. 1967). (c) Shortcut use differs among three human participant experiment groups, even though they are all able to physically point to the goal location (Wilson and Wilson 2018). (d) Human participants who select the paths adjacent to the training route (possible response strategy) do not self‐report lower spatial aptitude than participants who select the shortcut path (possible map‐based strategy) (Doner et al. 2023). (e) Left: in a variant of the Sunburst test, rats given a hippocampal lesion before training chose the shortcut less than control (‘Sham’) and cortically lesioned (‘Cortical’) rats. Right: the choices of rats given a hippocampal lesion after training do not differ from controls, with both groups preferring the shortcut more than chance (Harley 1979). Significance brackets give the result of chi‐square tests of expected proportions (Benjamini–Hochberg correction). (f) The error made in Sunburst path choice (° relative to the shortcut) is not correlated with the angular drift observed in thalamic head‐direction cells. (g) Left: when Doner et al. (2023) added distal cues to their apparatus, shortcut use did not change significantly (from 13.2% to 13.6%). Values shown are relative to chance. Text gives the result of a *z*‐difference test (difference in *z*‐scores minus the difference in shuffle means divided by the square root of summed shuffle variances). Right: comparing Young et al. (Group 1; 1967), who did not provide distal cues, to Gentry et al. (Experiment I, Group A; 1947), who provided a single distal cue, shortcut use did not differ significantly and was actually lower (15.0 vs. 0.0%).

Tolman (1948) argued that choosing the shortcut path in the Sunburst task reflected the use of a cognitive map. Yet, Tolman et al.'s (1946) animals took a remarkably long time to commit to a choice during the test phase, deliberating for an average of 3 min and 28 s, with no rat choosing in under 85 s (p. 18). Such prolonged hesitation is difficult to reconcile with the idea of a well‐formed cognitive map guiding rapid inference to the goal. Furthermore, rats selectively bred for superior maze learning (‘bright’ rats) performed no better on the shortcut task than those bred for poor maze performance (‘dull’ rats) (see Section 3.2): both groups chose the shortcut at equivalent rates (53% ‘brights’) (Figure 4a). This, coupled with evidence that non‐human primates do not seem to perform better in the task than rodents (Young et al. 1967) (Figure 4b), raises important questions about what ability the Sunburst maze is testing. In this section, we explore further evidence suggesting that performance in the task may not reflect a cognitive mapping ability or spatial navigation skill in the way Tolman (1948) originally proposed.

### 5.1 Performance Is Not Predictive of Spatial Knowledge

In a follow‐up test after Wilson and Wilson's (2018) Sunburst maze task, human participants were placed in the choice point of the maze and asked to point towards the reward location. The group of participants tested with a stable light source chose the shortcut path at the highest rate, and yet they performed no better in the pointing task than groups who did not choose the shortcut (Figure 4c). Similarly, participants who chose the shortcut path, presumably favouring a map‐based strategy, scored no differently on the Santa Barbara Sense of Direction scale (Hegarty 2002; Doner et al. 2023) than participants who chose adjacent paths and presumably favoured a response‐based strategy (Figure 4d). Lastly, participants asked to draw a map of the maze following testing produce reasonably accurate plan views regardless of their accuracy in the shortcut test (Wilson and Wilson 2018). These findings challenge the idea that humans and rats solve the Sunburst task using a cognitive map: If this was the case, shortcutting participants should also have exhibited higher spatial knowledge of the environment.

### 5.2 Shortcutting Preference Is Not Related to Brain Function

In one of the rare studies that found a shortcut preference, Harley (1979) examined the role of the hippocampus in a smaller and simplified variant of the Sunburst maze. Rats with hippocampal lesions made prior to training distributed their choices uniformly across the available arms (Figure 4e left ‘hippocampal’; Figure S3), while animals given posttraining hippocampal lesions chose the shortcut more than chance (Figure 4e right ‘hippocampal’; Figure S3c). This dissociation suggests that the hippocampus is involved in constructing a cognitive map (during training), which is necessary to infer a novel shortcut, but it is not involved in making a spatial inference or guiding shortcutting behaviour (during the test). This result supports Tolman's (1948) claim that the Sunburst task depends on a cognitive map, since the hippocampus is closely linked to spatial memory and navigation (O'Keefe and Nadel 1978; Squire 1992), particularly place navigation using distal cues (Morris et al. 1982; Packard and McGaugh 1996).

However, interpreting Harley's (1979) study is complicated by the fact that the shortcut path was also one of the paths adjacent to the training path (see Figure S3). Comparing across experiments, control rats did not reliably choose the shortcut path; instead, they favoured both paths adjacent to the trained path, suggesting a reliance on response‐based strategies (Section 4.2). For example, in Harley's (1979) first experiment, most rats (12/17 or 71%) chose the shortcut, which was also one of the adjacent paths, while the remaining rats chose the other adjacent path. In a second experiment with a different group of rats, only 15/34 (42%) chose the shortcut, while most chose the other adjacent path (Retention groups, pre‐op choices combined). As previously mentioned, Harley (1979) noted that, at the time of publication, 126 control rats had been tested in her Sunburst maze with more than 90% of them choosing ‘Arms 3 or 4’—the two adjacent paths, implying that rats did not favour the shortcut overall (p. 289). This difficulty in replicating the shortcutting effect within Harley's (1979) own study is consistent with the variability of the broader literature and weakens the case that shortcutting in the Sunburst task is dependent on the hippocampus or a cognitive map because it seems unlikely that the animals in this experiment were using a map‐based strategy.

In the only electrophysiological investigation of the Sunburst task, Muir and Taube (2004) were interested in how single neurons may inform an animal's choice of path in the test phase. They recorded the activity of head‐direction cells, a class of neurons that fire selectively when an animal's head is oriented in a specific direction, independent of location. These cells, found in brain regions such as the postsubiculum and anterior thalamus, are thought to underlie directional orientation using allocentric cues and support navigation based on self‐motion cues (Taube 1998, 2007; Yoder and Taube 2014). Muir and Taube (2004) tracked head‐direction cell activity as rats completed a smaller variant of the Sunburst maze (Figure S9a). The use of different training paths and reward locations allowed retesting of the same four animals, which was essential to properly quantify neural activity. Across 16 test sessions, these rats chose the shortcut path only once (6.25%, chance = 12.5%), while head‐direction cell activity remained consistent throughout all phases of the task, suggesting that this activity is not correlated with path choice in this task (Figure 4f; Figure S9b,c). For instance, in one case, a rat encountering the Sunburst maze for the first time immediately selected the novel shortcut path, and the preferred firing direction of its head‐direction cells remained stable compared to the training trials. In contrast, another rat that made seven incorrect choices before selecting the shortcut path also exhibited consistent head‐direction cell activity relative to the training trials. Across all animals, the average change in preferred firing direction of the head‐direction cells between the training and test trials was less than 1°, despite an average of 5.5 errors per animal (Muir and Taube 2004; Wiener and Taube 2005).

While this finding undermines the conclusion that shortcutting behaviour in the Sunburst maze results from a stable representation of one's direction, it does not directly test the role of the cognitive map in the task, which is thought to be implemented by hippocampal place cells (O'Keefe and Nadel 1978). To our knowledge, no study has recorded place cell activity in this maze. However, given the sensitivity of place cells to contextual changes (Anderson and Jeffery 2003) as well as changes in environmental geometry (O'Keefe and Burgess 1996; Grieves et al. 2016), one would expect clear remapping between the training and testing environments. With such an unstable map, it seems unlikely that place cells could support shortcutting in this task. It is however possible that grid cells, which tend to preserve their grid spacing and orientation across environments, might support it, via vector‐based navigation instead of map‐based shortcutting (Edvardsen et al. 2020).

### 5.3 Humans and Rodents Do Not use Distal Cues

Tolman et al. (1946) argued that distal cues, such as the light in their original experiment, are essential for animals to navigate to a specific spatial location, in contrast to response‐based navigation. Subsequent research has confirmed that place or ‘map‐based’ learning typically depends on integrating multiple distal cues (O'Keefe and Conway 1978; O'Keefe and Speakman 1987; Arleo and Rondi‐Reig 2007). However, Sunburst maze replications using both humans and rodent subjects suggest that distal cues do not play an important role. For example, Doner et al. (2023) found that adding distal cues to their virtual reality Sunburst maze did not increase the likelihood that participants chose the shortcut, suggesting that humans may rely on alternative strategies such as beaconing or vector‐based navigation rather than a true map‐based strategy (Figure 4g; Figure S4d). Similarly, in rodents, eliminating extramaze cues (Young et al. 1967) or manipulating light cues (Gentry et al. 1947; Birch and Korn 1958) had little effect: Rats still failed to choose the shortcut reliably, instead showing adjacency or directional biases (Figure 4g). Moreover, Tolman et al. (1946, 18) noted that when the training route was blocked, rats frequently returned to the start box before making a choice, behaviour more consistent with attempting to re‐execute a learned sequence of actions than computing a novel, map‐based route to the goal.

Together, these findings suggest that neither humans nor rodents typically solve the Sunburst task using a map‐based strategy, but instead depend on a mixture of procedural rules, cue‐based responses and apparatus‐driven biases. Indeed, Tolman et al. (1946) conducted their study in a plain room under low illumination: the exact type of environment which has since been shown to promote response and *not* place learning (Restle 1957; Packard and Goodman 2013).

## 6 Discussion

Throughout the 12 studies, comprising 46 experiments, that attempted to replicate Tolman et al.'s (1946) study in the ‘Sunburst maze’, only a few replicated the main effect of the original experiment. Because of this, the main conclusion—that rats can find shortcuts based on a cognitive map—should likely not be made from this specific study. While we have not found a unique factor that could explain the discrepancy between the results of the original study and its replication attempts, we discussed several peculiarities and limitations of the original design (e.g., rat strain, pretraining, quantification of visits, novelty of the test environment and presence of light cue next to reward location) that also alter confidence in the interpretation of the results. Results from replication attempts, instead of demonstrating preference for the shortcut path, either do not find any path preference or show a bias for the outer paths or the paths adjacent to the training route, highlighting either that rats had unclear expectations in the test environment, were using response‐based strategies, or thigmotaxis. Altogether, the main conclusion from this set of experiments is that the training‐then‐testing paradigm in two different mazes is not appropriate for robustly evidencing map‐based shortcutting. Importantly, however, this reinterpretation of Tolman et al.'s (1946) Sunburst maze does not challenge the broader cognitive map framework, which rests on multiple converging lines of behavioural, neurophysiological and theoretical evidence (O'Keefe and Nadel 1978; Morris 1981; Redish 1999). Rather, our analysis highlights that the Sunburst maze, long treated as a canonical demonstration of map‐based inference, does not provide a reliable behavioural signature of cognitive mapping. Future work will be essential to determine the specific forms of shortcutting or spatial inference that animals can achieve under well‐controlled conditions, and how these behaviours reflect the contributions of map‐based navigation, vector integration and other navigational strategies.

Following the publication of Tolman's influential review in 1948, interest in the cognitive map hypothesis waned, and relatively few studies addressed the topic until its revival in the 1970s with the publication of O'Keefe and Nadel's (1978) seminal work on the hippocampus. Tolman's collaborators continued their line of research through a series titled ‘Studies in Spatial Learning’, which concluded with papers by Ritchie et al. (1950), and Ritchie, Hay, and Hare (1951). These later studies took a more tempered stance, softening the bold claims of Tolman's original hypothesis. Importantly, they helped move the field beyond a theoretical impasse by suggesting that place learning and response learning need not be mutually exclusive—an idea that allowed for more flexible interpretations of spatial behaviour (Ellen and Thinus‐Blanc 1987). Building on Ritchie, Hay and Hare's (1951) findings, Restle (1957) proposed a theoretical framework in which the dominance of either learning strategy—place or response—depends on the environmental context. Specifically, he argued that richly cued environments are more likely to support the formation of cognitive maps, whereas sparse or featureless environments tend to promote response‐based strategies. Tolman et al.'s (1946) study holds an important position in psychological and neuroscience research and should be remembered and taught, but as Olton (1979) pointed out, ‘this experiment is often cited as a classic in support of cognitive mapping abilities in rats, such a choice is unfortunate … the results could not be replicated’ (p. 589).

We can, and should, work to correct the disconnect between the simplified version of Tolman et al.'s (1946) findings that appears in many introductory psychology lectures or textbooks and the contextualized picture revealed by detailed replication studies and modern analyses. Researchers should draw attention to these inaccuracies and point out where content misrepresents the evidence—such as portraying Tolman et al.'s (1946) rats as consistently taking shortcuts, without including the fact that numerous replications failed to find such behaviour. Finally, it is important to consider the likelihood of publication bias. Because null or negative findings are less likely to be published, the existing literature may under‐represent the true number of unsuccessful replications (Fanelli 2012; Song et al. 2013; de Vries et al. 2018). If these unpublished studies were taken into account, the overall pattern would likely shift further in favour of *no* shortcutting, making Tolman et al.'s (1946) findings appear as even more of an outlier relative to the wider literature. Taking the full picture into account, we can ensure that the next generation of psychologists and cognitive neuroscientists are taught not just the iconic experiments but also the complexities and controversies that make them worth studying.

## 7 Methods

### 7.1 Literature Search and Study Selection

We conducted a comprehensive literature search to identify studies examining shortcutting behaviour in rats or humans using the Tolman et al. (1946) ‘Sunburst’ maze. Searches were performed in PubMed, Google Scholar and ResearchRabbit, supplemented by backward and forward citation tracking. Textbooks, monographs, PhD theses and prior reviews were also consulted to ensure coverage of older studies. Studies were included if they employed the Sunburst maze or closely analogous tasks and reported empirical data on shortcutting. It should be noted that the available literature is likely biased towards positive findings, as ‘negative’ or ambiguous replications are less likely to have been published (Fanelli 2012; Song et al. 2013).

### 7.2 Data Extraction and Graph Digitization

Many of the identified studies were published using hand‐drawn graphs or with incomplete reporting of numerical data. To extract usable data, published graphs were digitized using the WebPlotDigitizer tool (v5.2, automeris.io). These digitized data are freely available (see Data Availability Statement). Two recent studies (Wilson and Wilson 2018; Doner et al. 2023) provided source data directly, which were used without digitization. For visualization, the mean ± SD values reported by Dawson (1972) were used to plot a probability density function (MATLAB *normpdf*) with the corresponding means and standard deviations.

### 7.3 Apparatus Reconstruction

To recreate maze schematics, we created figures using the scaled vector graphics programme Inkscape (v1.3.2, inkscape.org) based on reported dimensions and textual descriptions. This allowed for standardized representation of maze configurations across studies and facilitated additional analyses. These scaled vector graphics are freely available (see Data Availability Statement).

### 7.4 Random Path Choice Reallocation

In the included experiments, subjects typically selected one path from multiple alternatives (e.g., 18 paths) in a single trial. To determine whether subjects chose a particular path, such as a shortcut, more often than expected by chance, we implemented a resampling‐based approach.

For each experiment, we performed 1000 random resamples of N choices, with replacement (MATLAB *randi*), where *N* corresponded to the total number of observed choices made by the subjects. Each resample consisted of a random assignment of choices across available paths, and the resulting distribution was normalized to percentages. Across all resamples, we calculated the mean (*μ*_*r*_) and standard deviation (*σ*_*r*_) of path selections to generate a reference distribution under the null hypothesis of random choice.

Statistical thresholds for chance performance were defined using this reference distribution: the *p* = 0.05 threshold was calculated as *μ*_*r*_ + 1.650(*σ*_*r*_), and the *p* = 0.01 threshold was calculated as *μ*_*r*_ + 2.326(*σ*_*r*_). These values correspond to the *z*‐scores for a one‐sided normal distribution, representing the probability of observing a value as extreme or more extreme than the threshold by chance alone at the 5% and 1% levels, respectively. Observed path selection percentages exceeding these thresholds were considered significantly above chance. Where multiple groups are plotted using the same axis, for visualization only, the chance distribution for the first group listed in the legend is shown.

### 7.5 Chance Normalization (Z)

To allow comparison across experiments with different numbers of paths or subjects, observed path choice percentages were normalized relative to the reference distribution: normalized = (observed—*μ*_*r*_)/*σ*_*r*_. This normalization enabled standardized comparisons of shortcut selection across all included studies.

### 7.6 Experiment Categorization

After normalizing path choice percentages relative to chance (Methods: *Chance normalization (z)*) we categorized experiments according to the path type with the highest number of choices (e.g., shortcut, cued and outermost), this also had to be selected at an above chance rate (exceeding the chance distribution by more than 1.96 standard deviations). Choices that corresponded to more than one path (i.e., outermost and adjacent) were averaged. Where path types overlapped or were tied, we applied the rule: Shortcut > Adjacent > Outer > Cued > Other (i.e., equal choices of the shortcut and adjacent paths would be categorized as ‘shortcut’). Note that this rule biases results, wherever implemented, towards positive shortcutting. If no path type was chosen by subjects at an above chance rate, the experiment was categorized as having no preference (‘no pref.’). The resulting categorizations are used in Figure 3b.

For these categorizations we did not include all of the available data. In some studies, groups of rats were tested more than once (Harley 1979) or choices were scored in multiple ways (e.g., Birch and Korn 1958). For comparison to Tolman et al. (1946) and reporting of novel shortcutting, only first choice distributions were used. One exception to this was Young et al. (1967) who only reported mixed‐order choices without any further information; these mixed‐order choices were used. The hippocampal and cortical lesion groups of Harley (1979) were excluded as the animals were not intact. In the retention experiment of Harley (1979), all three groups (sham, hippocampal and cortical) were trained and then tested pre‐op, we combined these pre‐op values into one group as they represent a first Sunburst test on intact animals. Gentry et al.'s (1947) Experiment V groups and Kendler and Gasser's (1948) Group 0 were excluded because these animals received no training before the Sunburst test. The results of Dawson (1972) were not included because he did not provide path choice values, only the mean and standard deviation of path choices. Unpublished graduate thesis results were not included (Ritchie 1946; Chamberlain 1947; Michik 1973). The remaining data are the normalized choice distributions used in Figure 3c–e, these span 13 published studies and 47 unique experiments (including the original study).

## Author Contributions

**Éléonore Duvelle:** conceptualization, funding acquisition, investigation, methodology, visualization, writing – original draft, writing – review and editing. **Roddy M. Grieves:** conceptualization, data curation, formal analysis, funding acquisition, investigation, methodology, project administration, resources, software, supervision, validation, visualization, writing – original draft, writing – review and editing.

## Conflicts of Interest

The authors declare no conflicts of interest.

## Supporting information




**Figure S1:** Based on data/description from Tolman, Tryon and Jeffress (1929). (a) Left: the 17‐unit T‐maze used for selectively breeding the strains of rats used by Tolman (Section 3.2) and for pretraining them (Section 3.3). See also the figure provided by Munn (1951, 257, fig. 108) and the original provided by Tolman et al. (1929, 101) Figure 1. Animals learn to navigate from the ‘start’ position, available from their home cage mounted on a revolving ‘delivery table’, to the ‘end’ position where they enter a new cage above their previous one and receive food and water. While this is not specified, due to the constraints of moving such a maze in and out of a room, it is likely that the Sunburst maze and Tryon's maze were in different rooms. It is also likely, but not specified, that Tolman et al.'s (1946) rats followed the training procedure of the Tryon rats, which is as follows: for 7 days, rats are initially trained to use the P > T shortcut (dotted line), each day they are started progressively farther from the end point along this path and components of the maze (trapdoors, curtains, etc.) are added gradually. After this preliminary training, the shortcut is made inaccessible and the rat runs through the full T‐maze configuration. Rats take a ‘great amount of time’ on the first 2 days as they ‘explore the various alleys’, but this is ‘greatly reduced’ on the third day (Tolman et al. 1929, 112). Six days before Tolman et al.'s (1946) Sunburst maze experiment, their rats had completed 18 days (one trial per day) of training in this multiunit T‐maze (presumably not including the 7 days of pretraining). Right: Individual T‐maze component in more detail. Each unit contains a blind alley and an exit into the next unit. The dashed line in the figure indicates the path of a rat, which enters the unit at the bottom elbow piece, walks over the trap door, which moves up to prevent backtracking, comes to the choice point, enters the blind alley on the left (which would count as an error), returns and leaves the T‐component into the next unit. Black curtains were draped halfway along the blind and correct alleys so that the correct path cannot be determined visually. The maze was not cleaned between animals.
**Figure S2:** Based on data/description from Gentry et al. (1947) and Birch and Korn (1958). Chance calculated as described in Methods: *Random path choice reallocation*. (a) Maze configuration used. See also Gentry et al.'s (1947, 310) Figure 2. In Birch and Korn's (1958) experiment, test paths 13–18 were shorter than the others, replicating the design of Tolman et al. (1946 their Figure 1), but rats were allowed to choose these paths as their first choice. Note the two possible light locations, L1 and L2. Gentry et al. (1947) used two groups of rats: Group L1 was trained and tested with the light at position L1, group L2 with the light at L2. Birch and Korn (1958) used these and a third group of rats, L1&L2, who were trained and tested with the light at position L1 and L2. Rats were trained as in Tolman et al. (1946). (b) Results from Gentry et al. (1947). Final path choices made during testing as a percentage of choices made by all rats (each rat tested once). Values taken from Gentry et al. (1947, 313) their Figures 3 and 4. Rats used the adjacent paths and not the shortcut path. (c) Summary of further experiments, concentrating on the proportion of rats choosing the shortcut or adjacent paths (maximum). Again, rats used the adjacent paths and not the shortcut path. Chance as in b. (d–g) Results from Birch and Korn (1958). (d) Final path choices in the first Sunburst test. (e) Same, but showing all path entries. (f) Final path choices in a second Sunburst test after additional training. (g) Same, but showing all path entries.
**Figure S3:** Based on data/description from Harley (1979, 285). Chance calculated as described in Methods: *Random path choice reallocation*. Rats were tested multiple times in this variant of the Sunburst maze, but for comparison with other studies, only results from the first pre‐op (b) or first post‐op (c) test are shown. (a) Maze configuration used. Note that test path#4 is both an adjacent and shortcut path. Rats were given 50 training trials (8 per day for 6 days, plus 2 before the test trial). The entire maze was transparent, but alleyways ended in opaque curtains, passing through a curtain was scored as a choice. (b) Results for acquisition groups given brain lesions before training and testing. Final path choices made during testing as a percentage of choices made. (c) Same as b but for a different group of animals, trained and tested in the same way (retention, post‐op test and groups combined). (d) After 8 weeks, this second group of animals were then given brain lesions and tested a second time (postoperative test 1). Note that the previous Sunburst test was unrewarded.
**Figure S4:** Based on data/description from Wilson and Wilson (2018) and Doner et al. (2023). Chance calculated as described in Methods: *Random path choice reallocation*. (a) Maze configuration used. See also Wilson and Wilson (2018, 3) Figure 1 and Doner et al. (2023, 239) Figure 2. Note the different light sources that could be used during training (L1) and/or testing (L1–4). Wilson and Wilson (2018) tested three groups: One trained and tested with the light at position L1, a second trained at L1 but tested with the light moved to L2, and a third trained and tested under ambient illumination without a distinctive light source. Doner et al. (2023) extended this design with two further groups, trained with the light at L1 but tested with it relocated to L3 or L4, respectively. (b) Wilson and Wilson's (2018) results, final path choices made during testing as a percentage of participants making that choice. Values taken from their Figure 2 (p. 9). Participants tended to choose the paths adjacent to the training route, some used the shortcut route, some used the route heading to a light source in the opposite direction to the reward. (c–e) Doner et al.'s (2023) results, final path choices made during testing as a percentage of choices made. (c) Experiment 1, light sources were the only distal cues. Values taken from their Figure 3 (p. 245). Participants tended to choose the paths adjacent to the training route or heading to a light source. (d) Experiment 2, additional distal cues were provided, values taken from their Figure 5 (p. 250). Participants still tended to choose the paths adjacent to the training route or heading to a light source. (e) Experiment 3, using immersive virtual reality with treadmill, values taken from their Figure 7 (p. 256). Participants showed the same preferences as before but choices were also biased towards the direction of the reward.
**Figure S5:** Based on data/description from Young et al. (1967). Chance calculated as described in Methods: *Random path choice reallocation*. (a) Maze configuration used. See also their Figure 1 (p. 589). The maze was completely enclosed and illuminated from within. Animals were carried to the maze and placed inside without seeing external cues. Animals were trained until they made ‘consistent runs to the food box of under 1 min’. Note the two possible reward locations R1 and R2, animals were trained and tested with one of these. (b) Results for Groups 1, 2 and 4. Final path choices made during testing as a percentage of choices made. All 3 groups were trained with reward at R1. Group 1 (black) was composed of five rats. For Group 2 (red) the initial segment of the training path was completely removed during testing. Group 4 (green) was composed of blind animals. Values taken from their Figure 2 (p. 590). (c) Same as (b) but for experiment Group 3. This group was tested with Paths #6 and #7 (the adjacent paths) closed. (d) Same as (b) but for experiment Groups 5 and 6. Both groups were trained with reward at R2. Group 5 (blue) was composed of rats, while Group 6 (magenta) was composed of squirrel monkeys. Note: Young et al. (1967) do not specify which path is the shortcut; based on their Figure 1 (p. 589), Path 4 represents the closest path to R1 and Path 5 to R2.
**Figure S6:** Based on data/description from Ritchie (1948). Chance calculated as described in Methods: *Random path choice reallocation*. (a) Maze configuration. See also Ritchie's (1948, 662–663) Figure 3 and 4. Rats were trained and tested with lights above a rewarded location and an equivalent, opposite arm. For half of the rats the reward location was at the end of the left path (Group 1) and for the other half at the end of the right path (Group 2), but for visualization data have been regularized to match the conditions of Group 2. Between training and testing the start location moved to the opposite side of the maze, but the reward and light sources remained unchanged. Thus, a rat who was rewarded for turning left at the choice point during training must turn right at the choice point during the test. Rats were given 5 days of partial training (from locations within the maze to reward) followed by 2 days of eight daily trials from S to R. (b) Final path choices made during testing as a percentage of choices made. Values taken from Ritchie's (1948, 665) Figures 6 and 7. Rats did not use the shortcut path. Rats tended to choose the outermost path parallel to the training route that headed in the direction of the reward. (c) Same as (b) but showing all path entries. Rats did not exhibit a preference in exploring the outermost or shortcut paths.
**Figure S7:** Based on data/description from Kendler et al. (1948; 1949) and Chamberlain (1947). Chance calculated as described in Methods: *Random path choice reallocation*. (a) Maze configuration used. See also Kendler and Gasser (1948, 180) Figure 1. The light source is displayed here next to the maze but was positioned above the T‐maze choice point. Note the use of curtains to obscure the destination of each path in the test phase. For half of the rats the reward location was at the end of the left path and for the other half at the end of the right path, but for visualization, data have been regularized to match the conditions of the left group. Kendler and Gasser (1948) used four experimental groups that received 0, 5, 20 or 100 training trials (four trials per day), respectively. Kendler and Menchen's (1949) four experimental groups were given 6/6, 6/21, 21/6 and 21/21 h of food deprivation before training/test trials respectively, all animals received four training trials per day until they completed 20 correct trials. (b) Kendler and Gasser's (1948) results, final path choices made during testing as a percentage of choices made. Values taken from their Table 1 (p. 182). (c) 20 further trials after the test trial, number of times each path was chosen, group mean and standard deviation. Values taken from their Table 2 (p. 183). (d) Kendler and Menchen's (1949) results, final path choices made during testing as a percentage of choices made. Values taken from their Table 1 (p. 496). (e) 20 further trials after the test trial, number of times each path was chosen, group mean and standard deviation. Values taken from their Table 2 (p. 497).
**Figure S8:** Based on data/description from Gentry et al. (1948). Chance calculated as described in Methods: *Random path choice reallocation*. (a) Maze configuration used. See also their Figures 1 and 2 (p. 313–314). Note that the training route ends at a reward location on the opposite side of the choice platform from its beginning. Rats received one training trial a day for 11 days. (b) Final path choices made during testing as a percentage of choices made. Values extracted from Gentry et al.'s (1948, 316) Figure 4. Rats did not use the shortcut path. Rats tended to choose the paths adjacent to the training route, even though this headed away from the light cue and the reward location.
**Figure S9:** Based on data/description from Muir and Taube (2004). Chance calculated as described in Methods: *Random path choice reallocation*. (a) Top: maze configuration used that most closely resembles the one used by Tolman et al. (1946), see also their Figure 1. Note that no distal cues were provided and an enclosing curtain was used. Bottom: the three possible training configurations used (I–III) and their corresponding shortcut paths in the test phase (right). Before surgery, rats completed 5–10 training trials a day, with the maze in a training configuration, until they were able to complete five consecutive successful trials, each in less than 30s. After surgery, rats completed a further three of these training trials at least once a week. (b) Results for maze configuration II: this most closely resembles the one used by Tolman et al. (1946). Final path choices made during testing as a percentage of choices made, *N* = 6 choices, from two rats. Values extracted from Muir and Taube (2004, 250) Table 1. Rats chose the adjacent paths and not the shortcut path. (c) Summary of all three maze configurations, concentrating on the proportion of rats choosing the shortcut or adjacent paths (maximum). Chance as in (b).
**Figure S10:** Based on data/description from Dawson (1972) and Michik (1973). Only the mean and standard deviation of path choices were provided in these reports. While we show probability density functions (PDFs) based on the assumption of a normal distribution, the mean and standard deviation are not only a limited descriptor of a distribution, they are also inappropriate for ordinal data. Triangles denote the mean of each distribution. (a) Maze configuration used. Over 3 days the rats completed seven trials from the start to the reward. On Day 4 the rats were presented with the Sunburst test. Michik (1973) used a similar training protocol, although the arrangement of paths in his Sunburst test spanned more than 180°, with the outermost arms facing slightly back towards the start. Note that Dawson (1972) identified Path #5 as the shortcut, but based on his schematics and text descriptions the shortcut path was actually #6 as in Tolman et al. (1946); we show Path #6 as the shortcut. Note also that Dawson (1972) reported that the light was placed in ‘exactly the same position’ between the training and testing phases, but the position differs in his schematics by an even greater amount than Tolman et al.'s (1946) figures—the inconsistent positions shown in their schematics are reproduced here. (b) Results of Dawson's (1972) Trial 1 using control male and female ‘Sheffield white’ rats. (c) Same as (b) but for Trial 2 groups. The rats from Trial 1 were divided into groups. Experimental males were implanted with an oestrogen secreting pellet (methylstilbestrol, 12 mg), experimental females were implanted with a testosterone secreting pellet (methyltestosterone, 10 mg). Control animals remained unimplanted. (d) Same as (c) but for Trial 3 groups. The rats from Trial 2 were tested again 15 days later, in this time the oestrogen‐treated males were implanted with a second oestrogen pellet. (e) Results of Michik's (1973) experiment. Experimental male and female rats were fed a diet poor in protein (8%), control animals were fed a normal diet (27% protein).
**Table S1:** Overview of the main Sunburst studies. Dashes denote missing data. For comparison to Tolman et al. (1946) and reporting of novel shortcutting, only first choice results are shown. One exception is Young et al. (1967) who only reported mixed‐order choices without any further information. The hippocampal and cortical lesion groups of Harley (1979) were excluded and pre‐op values of her retention groups were combined (see Methods: *Experiment categorization*). Note that Gentry et al.'s (1947) experiment V and Kendler and Gasser's (1948) Group 0 received no training before the Sunburst test. The results of Dawson (1972) were not included because he did not provide path choice values, only the mean and standard deviation of path choices. Unpublished graduate thesis results were not included (Ritchie 1946; Chamberlain 1947; Michik 1973). Doner et al. (2018) and Wilson and Wilson (2018) experiment conditions: L‐L = stable light, A = ambient light, L‐BM = light underwent big move before testing, L‐SMR and L‐SML = light underwent small move right or left before testing, respectively. Boxes highlighted in green indicate variables very similar to the original study (75%–100% of the original study value); those highlighted in orange are somewhat, but not completely similar (50%–75% of original study value). For the choice scoring column, the values used for highlighting were the absolute distances the animals needed to travel.

## Data Availability

The summary data set, consisting of data from all replication experiments mentioned here, is available for download here: https://github.com/Neuroesc/Tolman_review_data.git. Matlab code is available for download which, in conjunction with the summary data set, can be used to regenerate all of the figures and analyses reported here: https://github.com/Neuroesc/Tolman_review_data.git.
