# Supplementary material for: Tolman's Sunburst Maze 80 Years on: A Meta‐Analysis Reveals Poor Replicability and Little Evidence for Shortcutting
Source: Eur J Neurosci. 2026 Jan 5;63(1):e70365. doi: 10.1111/ejn.70365 (PMC12766676; doi:10.1111/ejn.70365)
Supplement: Supplementary file 1 — Figure S1: Based on data/description from Tolman, Tryon and Jeffress (1929). (a) Left: the 17‐unit T‐maze used for selectively breeding the strains of rats used by Tolman (Section 3.2) and for pretraining them (Section 3.3). See also the figure provided by Munn (1951, 257, fig. 108) and the original provided by Tolman et al. (1929, 101) Figure 1. Animals learn to navigate from the ‘start’ position, available from their home cage mounted on a revolving ‘delivery table’, to the ‘end’ position where they enter a new cage above their previous one and receive food and water. While this is not specified, due to the constraints of moving such a maze in and out of a room, it is likely that the Sunburst maze and Tryon's maze were in different rooms. It is also likely, but not specified, that Tolman et al.'s (1946) rats followed the training procedure of the Tryon rats, which is as follows: for 7 days, rats are initially trained to use the P > T shortcut (dotted line), each day they are started progressively farther from the end point along this path and components of the maze (trapdoors, curtains, etc.) are added gradually. After this preliminary training, the shortcut is made inaccessible and the rat runs through the full T‐maze configuration. Rats take a ‘great amount of time’ on the first 2 days as they ‘explore the various alleys’, but this is ‘greatly reduced’ on the third day (Tolman et al. 1929, 112). Six days before Tolman et al.'s (1946) Sunburst maze experiment, their rats had completed 18 days (one trial per day) of training in this multiunit T‐maze (presumably not including the 7 days of pretraining). Right: Individual T‐maze component in more detail. Each unit contains a blind alley and an exit into the next unit. The dashed line in the figure indicates the path of a rat, which enters the unit at the bottom elbow piece, walks over the trap door, which moves up to prevent backtracking, comes to the choice point, enters the blind alley on the left (which would cou [file EJN-63-0-s001.pdf]

Supplementary Figures

a Tolman, Tryon and Jeffress (1929) maze configuration

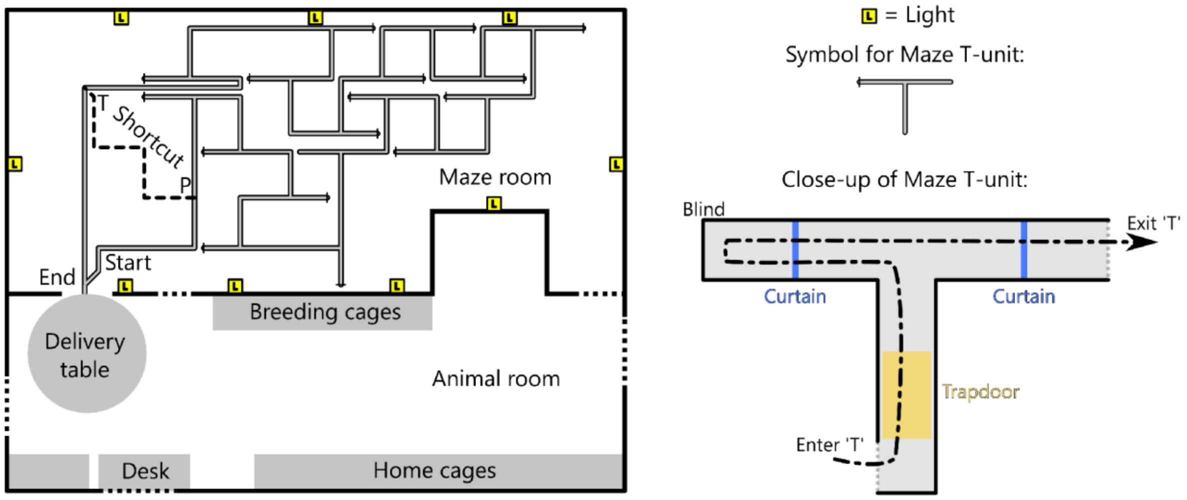

Fig. S1: Based on data/description from Tolman, Tryon and Jeffress (1929). **(a)** Left: the 17-unit T-maze used for selectively breeding the strains of rats used by Tolman (section 3.2) and for pretraining them (section 3.3). See also the figure provided by Munn (1951 p. 257, Fig. 108) and the original provided by Tolman et al. (1929 p. 101) Fig. 1. Animals learn to navigate from the 'start' position, available from their home cage mounted on a revolving 'delivery table', to the 'end' position where they enter a new cage above their previous one and receive food and water. While this is not specified, due to the constraints of moving such a maze in and out of a room, it is likely that the Sunburst maze and Tryon's maze were in different rooms. It is also likely, but not specified, that Tolman et al.'s (1946) rats followed the training procedure of the Tryon rats, which is as follows: for 7 days, rats are initially trained to use the P>T shortcut (dotted line), each day they are started progressively farther from the end point along this path and components of the maze (trapdoors, curtains etc) are added gradually. After this preliminary training, the shortcut is made inaccessible and the rat runs through the full T-maze configuration. Rats take a "great amount of time" on the first two days as they "explore the various alleys" but this is "greatly reduced" on the third day (Tolman *et al.*, 1929 p. 112)(Tolman *et al.*, 1929 p. 112)(Tolman *et al.*, 1929 p. 112). Six days before Tolman et al.'s (1946) Sunburst maze experiment, their rats had completed 18 days (one trial per day) of training in this multi-unit T-maze (presumably not including the 7 days of pretraining). Right: Individual T-maze component in more detail. Each unit contains a blind alley and an exit into the next unit. The dashed line in the figure indicates the path of a rat which enters the unit at the bottom elbow piece, walks over the trap door which moves up to prevent backtracking, comes to the choice point, enters the blind alley on the left (which would count as an error), returns and leaves the T-component into the next unit. Black curtains were draped halfway along the blind and correct alleys so that the correct path cannot be determined visually. The maze was not cleaned between animals.

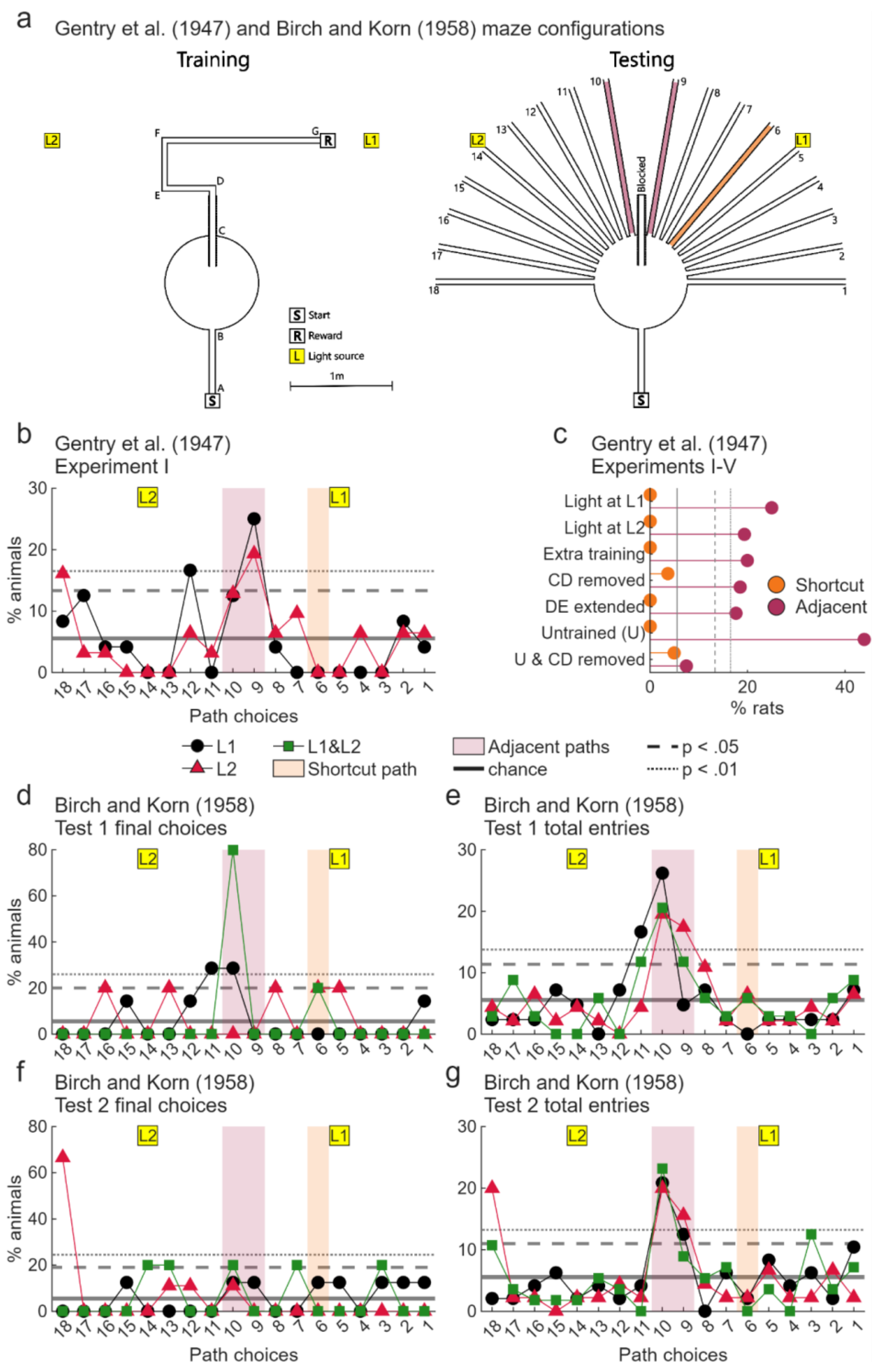

Fig. S2: Based on data/description from Gentry et al. (1947) and Birch and Korn (1958)(1958). Chance calculated as described in Methods: _Random path choice reallocation_. **(a)** Maze configuration used. See also Gentry et al.'s (1947 p. 310) Fig 2. In Birch and Korn's (1958) experiment, test paths 13-18 were shorter than the others, replicating the design of Tolman et al. (1946 their Fig 1), but rats were allowed to choose these paths as their first choice. Note the two possible light locations, L1 and L2. Gentry et al. (1947) used two groups of rats: group L1 was trained and tested with the light at position L1, group L2 with the light at L2. Birch and Korn (1958) used these and a third group of rats, L1&L2, who were trained and tested with the light at position L1 and L2. Rats were trained as in Tolman et al. (1946). **(b)** Results from Gentry et al. (1947). Final path choices made during testing as a percentage of choices made by all rats (each rat tested once). Values taken from Gentry et al. (1947 p. 313) their Fig 3 & 4. Rats used the adjacent paths and not the shortcut path. **(c)** Summary of further experiments, concentrating on the proportion of rats choosing the shortcut or adjacent paths (maximum). Again, rats used the adjacent paths and not the shortcut path. Chance as in b. **(d-g)** Results from Birch and Korn (1958). **(d)** Final path choices in the first Sunburst test. **(e)** Same, but showing all path entries. **(f)** Final path choices in a second Sunburst test after additional training. **(g)** Same, but showing all path entries.

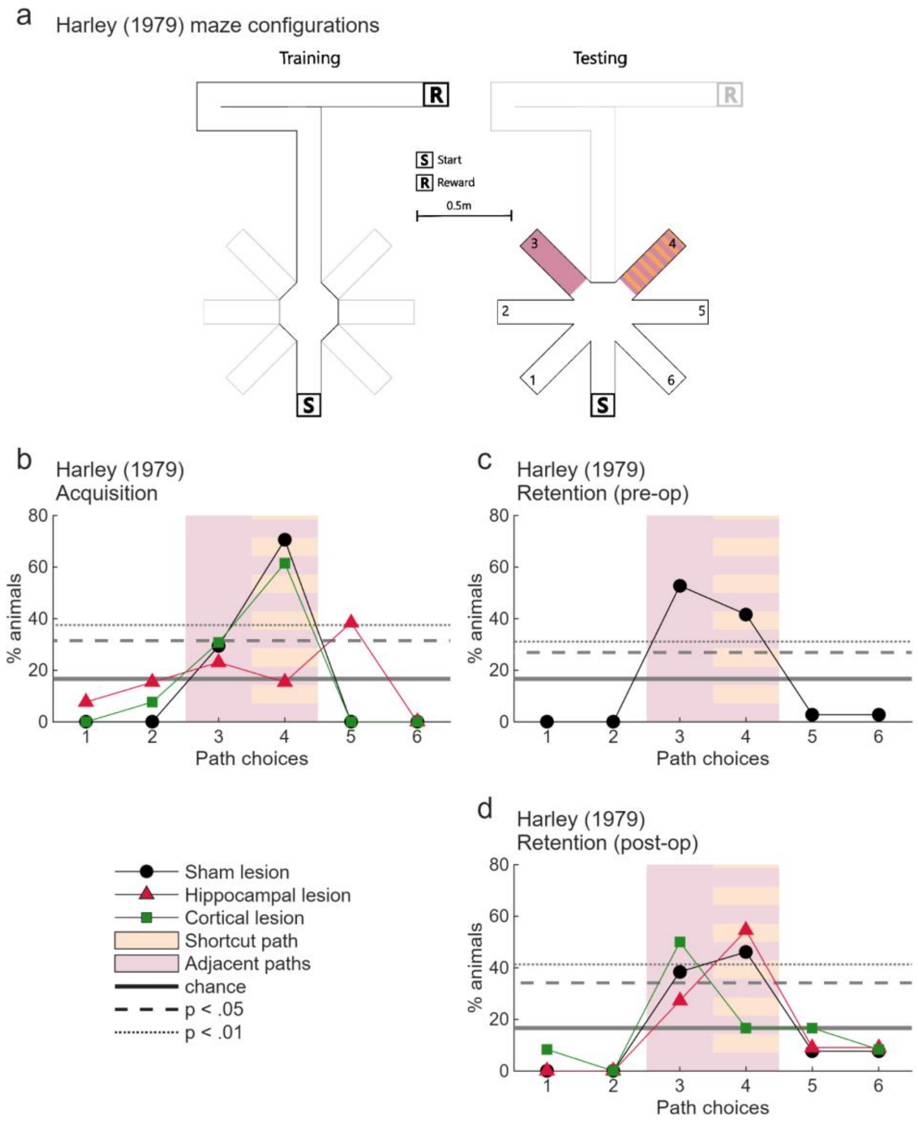

Fig. S3: Based on data/description from Harley (1979 p. 285). Chance calculated as described in Methods: *Random path choice reallocation*. Rats were tested multiple times in this variant of the Sunburst maze, but for comparison with other studies, only results from the first preop (b) or first post-op (c) test are shown. **(a)** Maze configuration used. Note that test path#4 is both an adjacent and shortcut path. Rats were given 50 training trials (8 per day for 6 days, plus 2 before the test trial). The entire maze was transparent, but alleyways ended in opaque curtains, passing through a curtain was scored as a choice. **(b)** Results for acquisition groups given brain lesions before training and testing. Final path choices made during testing as a percentage of choices made. **(c)** Same as b but for a different group of animals, trained and tested in the same way (retention, post-op test, groups combined). **(d)** After 8 weeks, this second group of animals were then given brain lesions and tested a second time (postoperative test 1). Note that the previous Sunburst test was unrewarded.

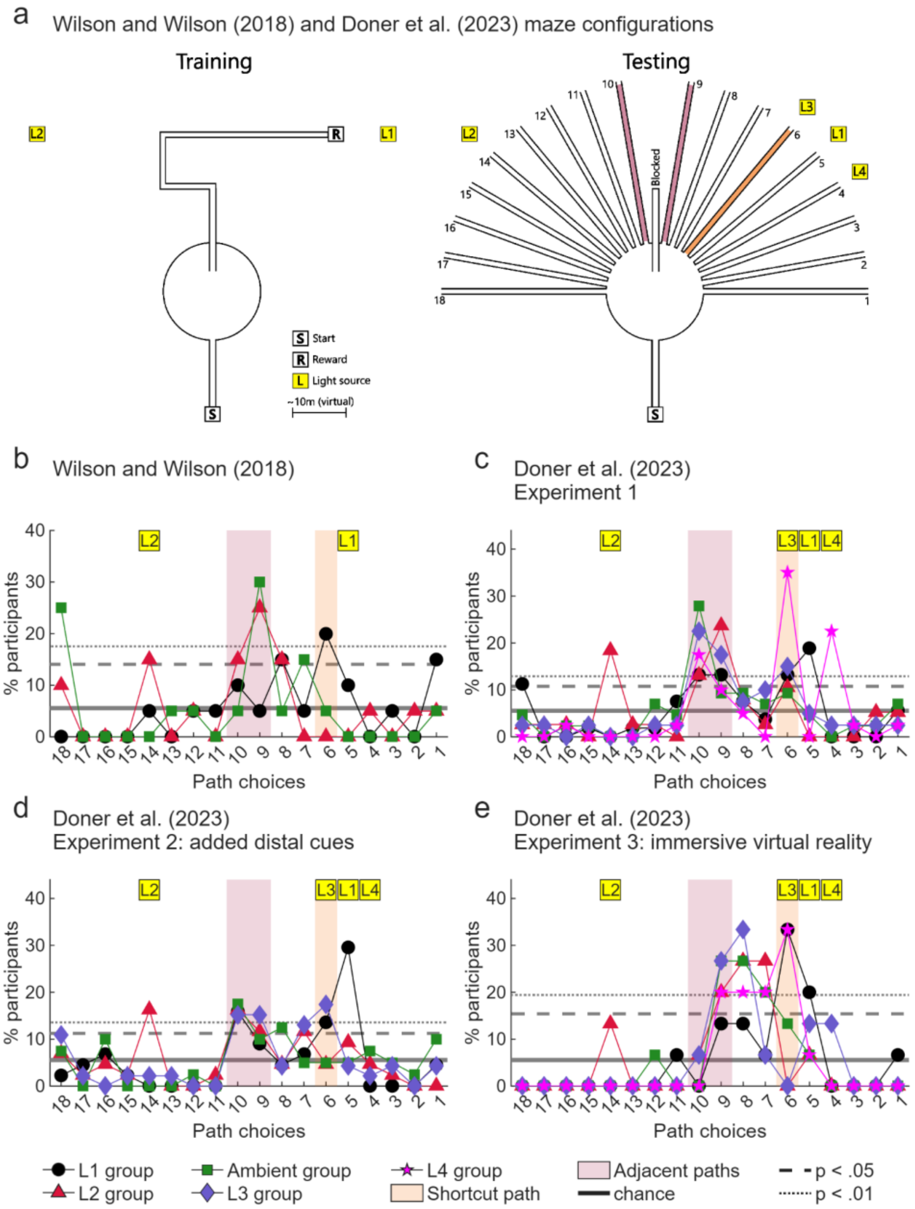

Fig. S4**:** Based on data/description from Wilson and Wilson (2018) and Doner et al. (2023). Chance calculated as described in Methods: *Random path choice reallocation*. **(a)** Maze configuration used. See also Wilson and Wilson (2018 p. 3) Fig 1 & Doner et al. (2023 p. 239) Fig 2. Note the different light sources that could be used during training (L1) and/or testing (L1-4). Wilson and Wilson (2018) tested three groups: one trained and tested with the light at position L1, a second trained at L1 but tested with the light moved to L2, and a third trained and tested under ambient illumination without a distinctive light source. Doner et al. (2023) extended this design with two further groups, trained with the light at L1 but tested with it relocated to L3 or L4, respectively. **(b)** Wilson and Wilson's (2018) results, final path choices made during testing as a percentage of participants making that choice. Values taken from their Fig 2 (p. 9). Participants tended to choose the paths adjacent to the training route, some used the shortcut route, some used the route heading to a light source in the opposite direction to the reward. **(c-e)** Doner et al.'s (2023) results, final path choices made during testing as a percentage of choices made. **(c)** Experiment 1, light sources were the only distal cues. Values taken from their Fig 3 (p. 245). Participants tended to choose the paths adjacent to the training route or heading to a light source. **(d)** Experiment 2, additional distal cues were provided, values taken from their Fig 5 (p. 250). Participants still tended to choose the paths adjacent to the training route or heading to a light source. **(e)** Experiment 3, using immersive virtual reality with treadmill, values taken from their Fig 7 (p. 256). Participants showed the same preferences as before but choices were also biased towards the direction of the reward.

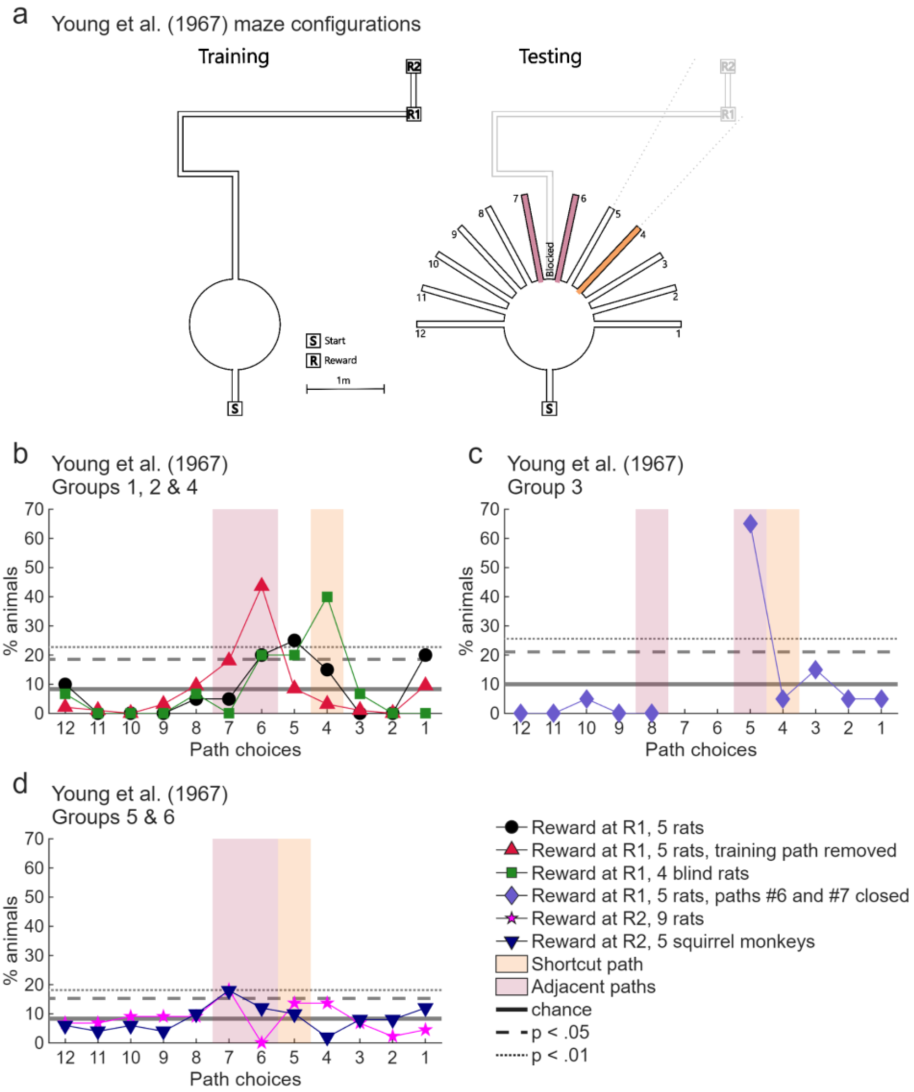

Fig. S5: Based on data/description from Young et al. (1967). Chance calculated as described in Methods: *Random path choice reallocation*. **(a)** Maze configuration used. See also their Fig 1 (p. 589). The maze was completely enclosed and illuminated from within. Animals were carried to the maze and placed inside without seeing external cues. Animals were trained until they made "consistent runs to the food box of under 1 min". Note the two possible reward locations R1 and R2, animals were trained and tested with one of these. **(b)** Results for groups 1, 2 and 4. Final path choices made during testing as a percentage of choices made. All 3 groups were trained with reward at R1. Group 1 (black) was composed of 5 rats. For group 2 (red) the initial segment of the training path was completely removed during testing. Group 4 (green) was composed of blind animals. Values taken from their Fig 2 (p. 590). **(c)** Same as b but for experiment group 3. This group was tested with paths #6 and #7 (the adjacent paths) closed. **(d)** Same as b but for experiment groups 5 and 6. Both groups were trained with reward at R2. Group 5 (blue) was composed of rats, while group 6 (magenta) was composed of squirrel monkeys. Note: Young et al. (1967) do not specify which path is the shortcut; based on their Fig 1 (p. 589), path 4 represents the closest path to R1 and path 5 to R2.

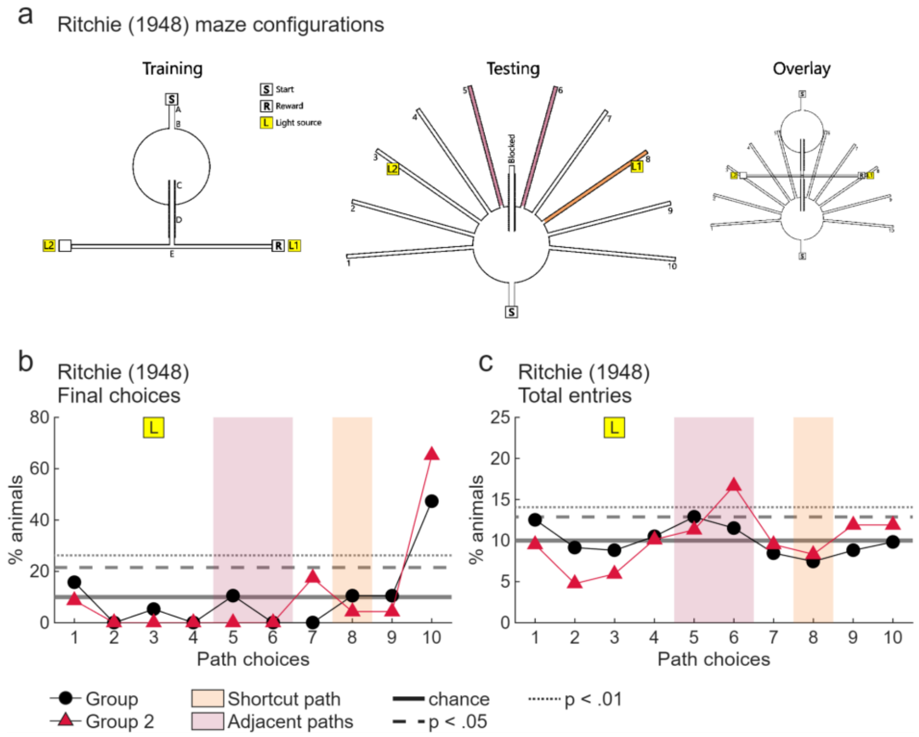

Fig. S6**:** Based on data/description from Ritchie (1948). Chance calculated as described in Methods: *Random path choice reallocation*. **(a)** Maze configuration. See also Ritchie's (1948 p. 662-663) Fig 3 & 4. Rats were trained and tested with lights above a rewarded location and an equivalent, opposite arm. For half of the rats the reward location was at the end of the left path (Group 1) and for the other half at the end of the right path (Group 2), but for visualisation data have been regularised to match the conditions of Group 2. Between training and testing the start location moved to the opposite side of the maze, but the reward and light sources remained unchanged. Thus, a rat who was rewarded for turning left at the choice point during training must turn right at the choice point during the test. Rats were given 5 days of partial training (from locations within the maze to reward) followed by 2 days of 8 daily trials from S to R. **(b)** Final path choices made during testing as a percentage of choices made. Values taken from Ritchie's (1948 p. 665) Fig 6 & 7. Rats did not use the shortcut path. Rats tended to choose the outermost path parallel to the training route that headed in the direction of the reward. **(c)** Same as b but showing all path entries. Rats did not exhibit a preference in exploring the outermost or shortcut paths.

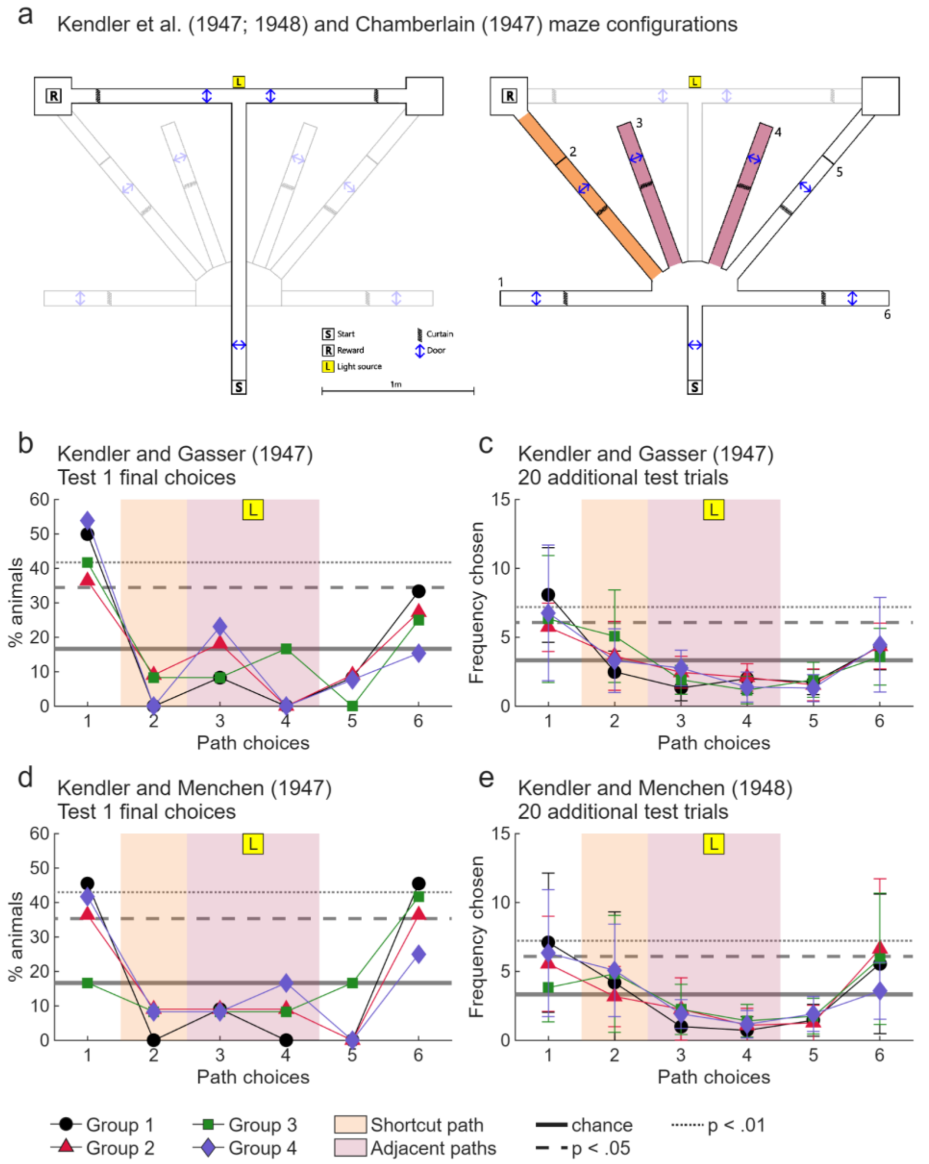

Fig. S7: Based on data/description from Kendler et al. (1948; 1949) and Chamberlain (1947). Chance calculated as described in Methods: *Random path choice reallocation*. **(a)** Maze configuration used. See also Kendler and Gasser (1948 p. 180) Fig 1. The light source is displayed here next to the maze, but was positioned above the T-maze choice point. Note the use of curtains to obscure the destination of each path in the test phase. For half of the rats the reward location was at the end of the left path and for the other half at the end of the right path, but for visualisation data have been regularised to match the conditions of the left group. Kendler and Gasser (1948) used 4 experimental groups that received 0, 5, 20 or 100 training trials (4 trials per day), respectively. Kendler and Menchen's (1949) 4 experimental groups were given 6/6, 6/21, 21/6, 21/21 hours of food deprivation before training/test trials respectively, all animals received 4 training trials per day until they completed 20 correct trials. **(b)** Kendler and Gasser's (1948) results, final path choices made during testing as a percentage of choices made. Values taken from their Table 1 (p. 182). **(c)** 20 further trials after the test trial, number of times each path was chosen, group mean and standard deviation. Values taken from their Table 2 (p. 183). **(d)** Kendler and Menchen's (1949) results, final path choices made during testing as a percentage of choices made. Values taken from their Table 1 (p. 496). **(e)** 20 further trials after the test trial, number of times each path was chosen, group mean and standard deviation. Values taken from their Table 2 (p. 497).

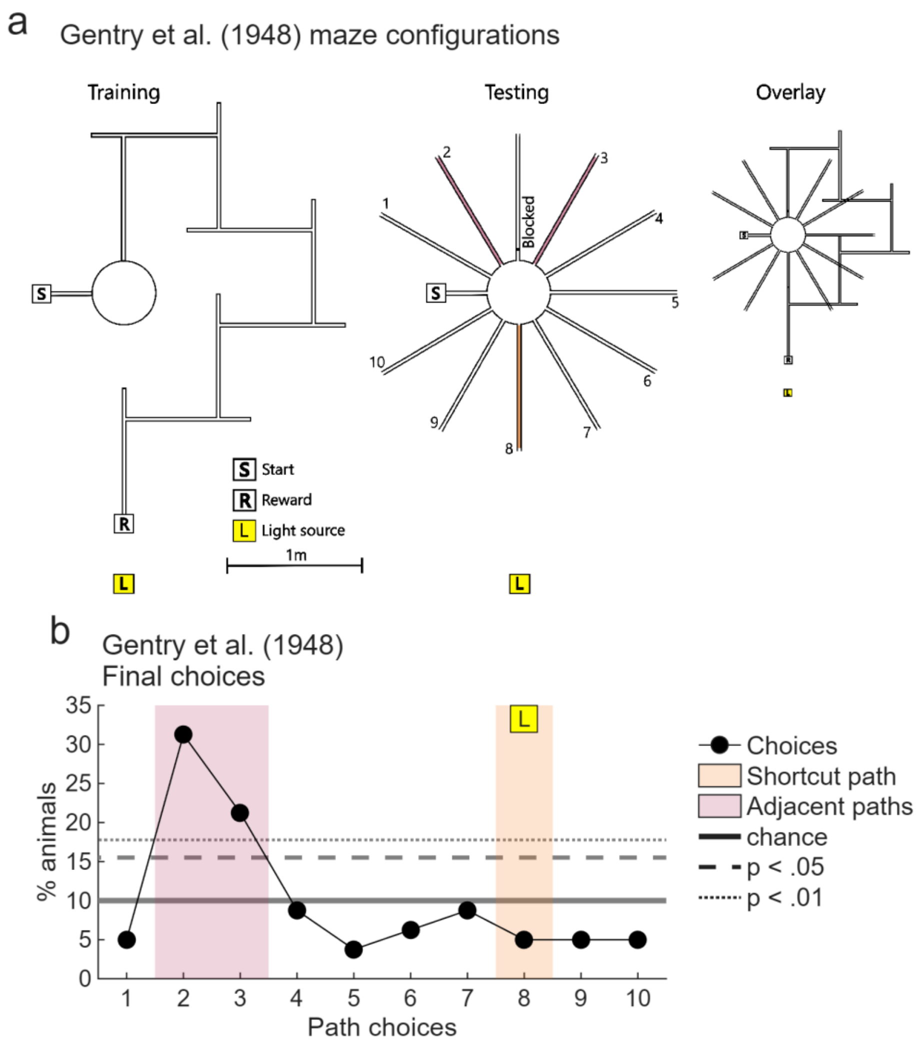

Fig. S8**:** Based on data/description from Gentry et al. (1948). Chance calculated as described in Methods: *Random path choice reallocation*. **(a)** Maze configuration used. See also their Fig 1 & 2 (p. 313-314). Note that the training route ends at a reward location on the opposite side of the choice platform from its beginning. Rats received 1 training trial a day for 11 days. **(b)** Final path choices made during testing as a percentage of choices made. Values extracted from Gentry et al.'s (1948 p. 316) Fig 4. Rats did not use the shortcut path. Rats tended to choose the paths adjacent to the training route, even though this headed away from the light cue and the reward location.

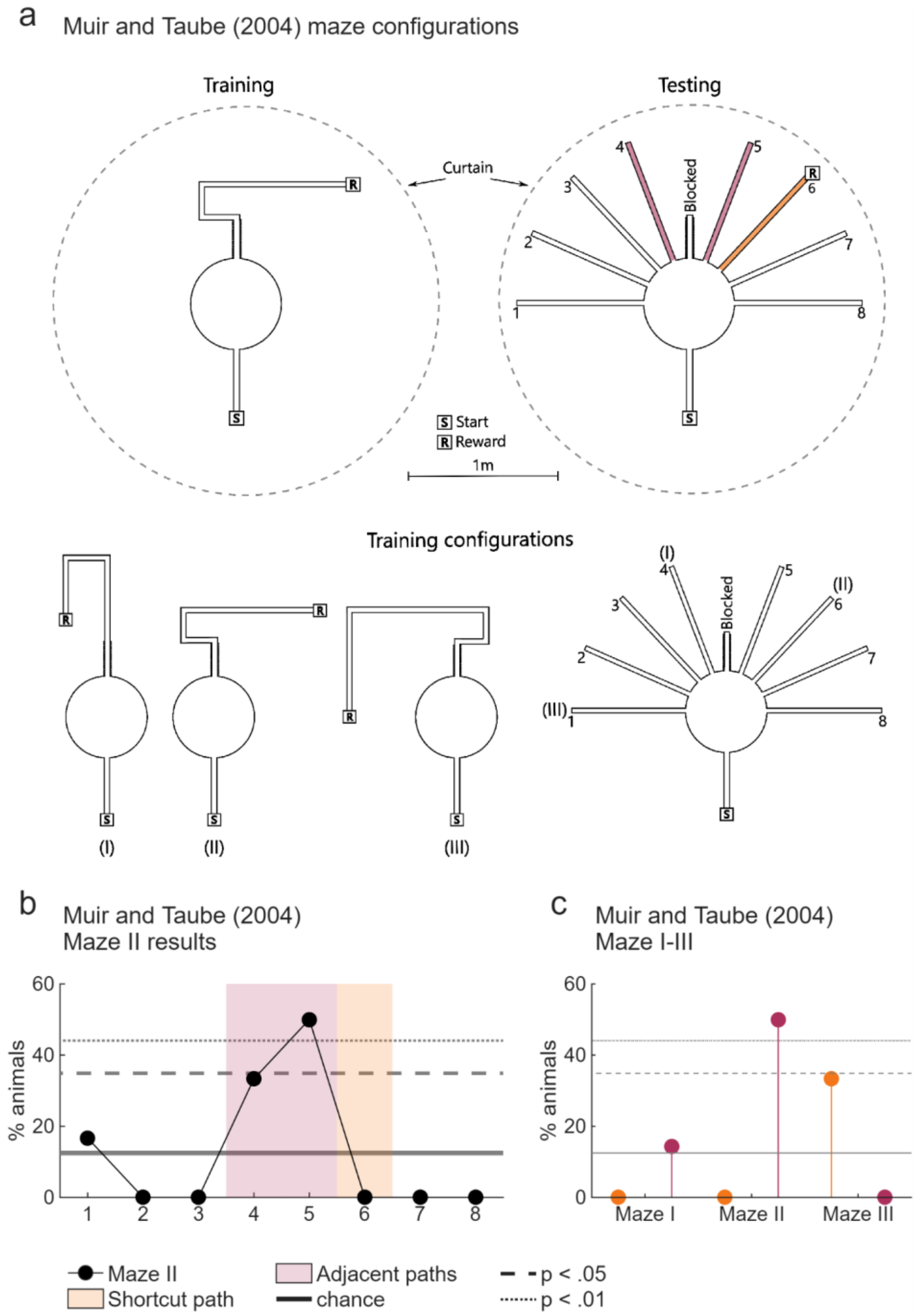

Fig. S9**:** Based on data/description from Muir and Taube (2004). Chance calculated as described in Methods: *Random path choice reallocation*. **(a)** Top: maze configuration used that most closely resembles the one used by Tolman et al. (1946), see also their Fig. 1. Note that no distal cues were provided and an enclosing curtain was used. Bottom: the three possible training configurations used (I-III) and their corresponding shortcut paths in the test phase (right). Before surgery, rats completed 5-10 training trials a day, with the maze in a training configuration, until they were able to complete five consecutive successful trials, each in less than 30s. After surgery, rats completed a further 3 of these training trials at least once a week. **(b)** Results for maze configuration II: this most closely resembles the one used by Tolman et al. (1946). Final path choices made during testing as a percentage of choices made, N = 6 choices, from 2 rats. Values extracted from Muir and Taube (2004 p. 250) Table 1. Rats chose the adjacent paths and not the shortcut path. **(c)** Summary of all 3 maze configurations, concentrating on the proportion of rats choosing the shortcut or adjacent paths (maximum). Chance as in b.

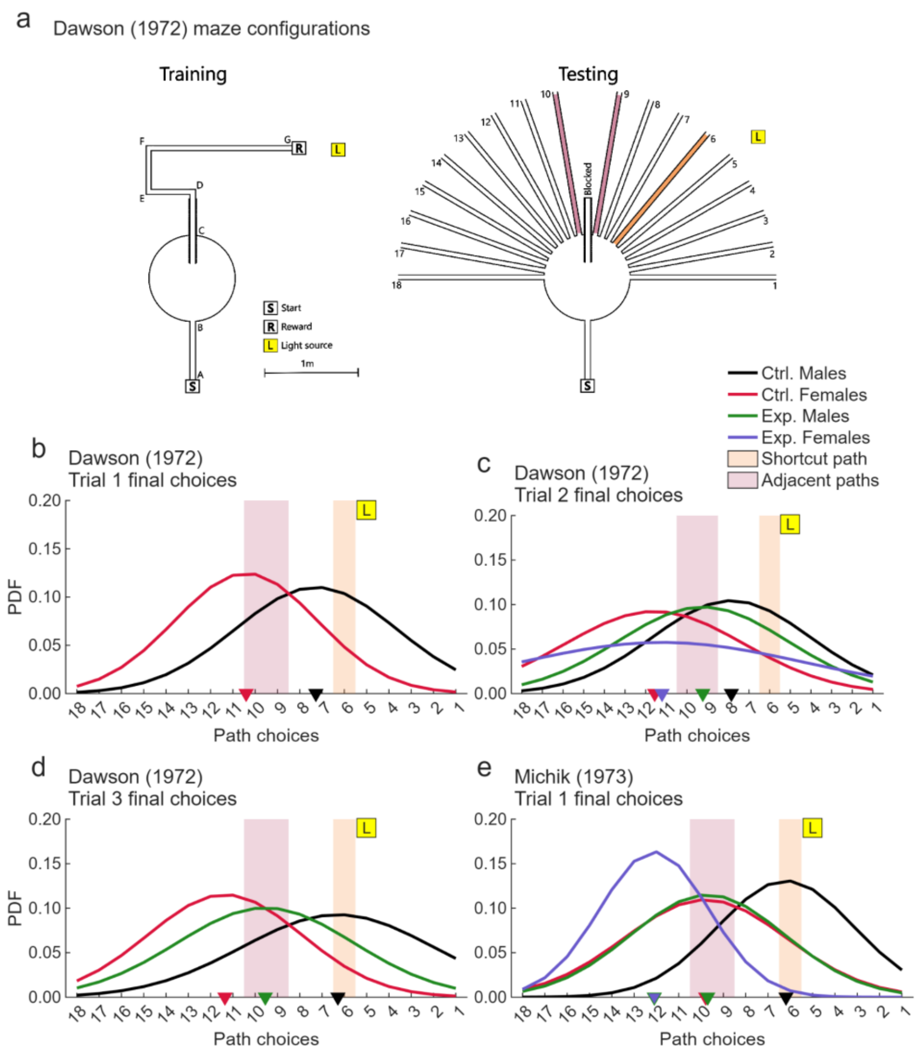

Fig. S10: Based on data/description from Dawson (1972) and Michik (1973). Only the mean and standard deviation of path choices were provided in these reports. While we show probability density functions (PDFs) based on the assumption of a normal distribution, the mean and standard deviation are not only a limited descriptor of a distribution, they are also inappropriate for ordinal data. Triangles denote the mean of each distribution. **(a)** Maze configuration used. Over 3 days the rats completed 7 trials from the start to the reward. On day 4 the rats were presented with the Sunburst test. Michik (1973) used a similar training protocol, although the arrangement of paths in his Sunburst test spanned more than 180°, with the outermost arms facing slightly back towards the start. Note that Dawson (1972) identified path #5 as the shortcut, but based on his schematics and text descriptions the shortcut path was actually #6 as in Tolman et al. (1946); we show path #6 as the shortcut. Note also that Dawson (1972) reported that the light was placed in "exactly the same position" between the training and testing phases, but the position differs in his schematics by an even greater amount than Tolman et al.'s (1946) figures - the inconsistent positions shown in their schematics are reproduced here. **(b)** Results of Dawson's (1972) Trial 1 using control male and female "Sheffield white" rats. **(c)** Same as b but for Trial 2 groups. The rats from Trial 1 were divided into groups. Experimental males were implanted with an estrogen secreting pellet (methylstilbestrol, 12 mg), experimental females were implanted with a testosterone secreting pellet (methyltestosterone, 10 mg). Control animals remained unimplanted. **(d)** Same as c but for Trial 3 groups. The rats from Trial 2 were tested again 15 days later, in this time the estrogen-treated males were implanted with a second estrogen pellet. **(e)** Results of Michik's (1973) experiment. Experimental male and female rats were fed a diet poor in protein (8%), control animals were fed a normal diet (27% protein).

Study	Experiment	Group	Apparatus: Choice scoring (% of distance)	Left arms excluded	New arms added in test	Cue near goal	Cleaning between subjects	Subjects: Species & strain	Pigmented	Age (weeks)	Sex	Pre-training	N	Results: % that made a choice	% shortcut use
**Tolman et al.** (1946)			100% of 1.83 m	Y	Y	Y	Choice platform rotated	Tryon (S_1 and S_3)	Y	~13 weeks	F	Tryon maze (18 trials)	56	94.6	36
**Gentry et al.** (1947)	I	A	100% of 1.83 m	N	Y	Y	Choice platform rotated	Wistar × albino	N	13-22 weeks	-	Untrained	27	88.9	0.0
		B				Y						H-type maze	22	90.9	4.5
		C				N							22	95.5	0.0
	II					Y						Untrained	22	95.4	0.0
	III												27	100	3.7
	IV												18	100	0.0
	V												103	87.4	0.0
**Ritchie** (1948)			80% of 1.5 m	N	Y	Y & opposite	-	Tryon (M×M)	Y	~13 weeks	M	Untrained	50	84	7.1
**Kendler and Gasser** (1948)		0	50% of 1.5 m	N	Present but blocked	N	Choice compartment wiped with damp cloth	Wistar	N	~10 weeks	M	Untrained	12	100	0.0
		5											12	100	9.1
		20											12	100	8.3
		100											12	100	0.0
**Gentry et al.** (1948)			100% of 0.92 m	N	Y	Y	Maze was dismantled daily and washed	Wistar & Hooded	mix ed	14-21 weeks	-	Untrained	80	100	5.0
**Kendler and Menchen** (1949)		6-6	50% of 1.5 m	N	Present but blocked	N	Choice compartment and 1ft of each alley wiped with damp cloth	Wistar	N	10 weeks	M	Untrained	11	100	0.0
		6-21											11	100	9.1
		21-6											12	100	8.3
		21-21											12	100	8.3
**Birch and Korn** (1958)		1	100% of 1.83 m	Y	Y	Y	Choice platform rotated	Sherman	N	30-35 weeks	F	14 days in a "practice elevated maze"	12	58.3	0.0
		2				N							12	41.7	20.0
		3				Y & opposite							12	41.7	20.0
**Young et al.** (1967)		1	-	N	Present but blocked	N	-	Unspecified albino	N	-	-	-	5	-	15.0
		2											5	-	3.2
		3											5	-	5.0
		4											4	-	40.0
		5											9	-	13.6
		6						Squirrel monkeys *(Saimiri sciureus)*					5	-	10.0

**Dawson** (1972)		M	16% of 1.83 m	N	Y	Y	Paths were rotated, possibly the choice platform as well	“Sheffield white”	-	>14 weeks	M	6 days in a Hebb-Williams (1946) maze	30	86.7	-
		F									F		30	90	-
**Harley** (1979)	Acquisition	Sham	100% of 0.4 m	N	Present but blocked	N	-	Sprague-Dawley	Y	13-35 weeks	M	-	47	91.5	70.6
	Retention	Sham											36	100	41.7
**Muir and Taube** (2004)			100% of 0.92 m	N	Y	N	-	Long-Evans	N	13-17 weeks	F	Foraging in a cylinder	4	n/a	8.3
**Wilson and Wilson** (2018)		L-L	-	N	Y	Y	Virtual reality	Human		Mean ± s.d.: 20.33 ± 0.97 years	mixed	VR familiarisation	10 M, 10 F	100	20.0
		A				N							10 M, 10 F	100	5.0
		L-BM				Moved in testing							10 M, 10 F	100	0.0
**Doner et al.** (2023)	1	A	100% of 64 m	N	Y	N	Virtual reality	Human		18-52 years, mean: 19.8	mixed	VR familiarisation	15 M, 27 F, 1 other	100	9.3
		L-L				Y							18 M, 35 F	100	13.2
		L-BM				Moved in testing							12 M, 24 F, 1 other	100	10.5
		L-SMR											10 M, 30 F	100	35.0
		L-SML											12 M, 28 F	100	15.0
	2	A				N				18-32 years, mean: 19.1			9 M, 31 F, 3 other	100	5.0
		L-L				Y							9 M, 27 F, 4 other	100	13.6
		L-BM				Moved in testing							18 M, 24 F, 1 other	100	5.0
		L-SMR											13 M, 32 F	100	17.4
	3	A				N				18-25 years, mean: 20.9			8 M, 10 F	100	13.3
		L-L				Y									33.3
		L-BM				Moved in testing									0.0
		L-SMR													33.3
		L-SML													0.0

Table S1: Overview of the main Sunburst studies. Dashes denote missing data. For comparison to Tolman et al. (1946) and reporting of novel shortcutting, only first choice results are shown. One exception is Young et al. (1967) who only reported mixed-order choices without any further information. The hippocampal and cortical lesion groups of Harley (1979) were excluded and pre-op values of her retention groups were combined (see Methods: *Experiment categorisation*). Note that Gentry et al.’s (1947) experiment V and Kendler and Gasser’s (1948) Group 0 received no training before the Sunburst test. The results of Dawson (1972) were not included because he did not provide path choice values, only the mean and standard deviation of path choices. Unpublished graduate thesis results were not included (Ritchie, 1946; Chamberlain, 1947; Michik, 1973). Doner et al. (2018) and Wilson and Wilson (2018) experiment conditions: L-L = stable light, A = ambient light, L-BM = light underwent big move before testing, L-SMR and L-SML = light underwent small move right or left before testing respectively. Boxes highlighted in green indicate variables very similar to the original study (75-100% of the original study value); those highlighted in orange are somewhat, but not completely similar (50-75% of original study value). For the choice scoring column, the values used for highlighting were the absolute distances the animals needed to travel.
